# The efficacy of neoadjuvant immunotherapy and lymphocyte subset predictors in locally advanced esophageal squamous cell carcinoma: A retrospective study

**DOI:** 10.1002/cam4.70228

**Published:** 2024-09-14

**Authors:** Ruotong Wang, Shaodi Wen, Xiaoyue Du, Jingwei Xia, Bowen Hu, Yihan Zhang, Guoren Zhou, Feng Jiang, Xiaomin Lu, Miaolin Zhu, Xinyu Xu, Bo Shen

**Affiliations:** ^1^ Department of Oncology, The Affiliated Cancer Hospital of Nanjing Medical University, Jiangsu Cancer Hospital Jiangsu Institute of Cancer Research Nanjing China; ^2^ Department of Thoracic Surgery, The Affiliated Cancer Hospital of Nanjing Medical University, Jiangsu Cancer Hospital Jiangsu Institute of Cancer Research Nanjing China; ^3^ Department of Oncology Affiliated Haian Hospital of Nantong University Haian Nantong China; ^4^ Department of Pathology, The Affiliated Cancer Hospital of Nanjing Medical University, Jiangsu Cancer Hospital Jiangsu Institute of Cancer Research Nanjing China; ^5^ Department of Oncology, Huaian Hospital of Huaian City Huaian Cancer Hospital Huaian China

**Keywords:** biomarkers, esophageal squamous cell, immunology, neoadjuvant chemotherapy, nomogram, prognostic factor

## Abstract

**Background:**

Despite the recognized therapeutic potential of programmed cell death protein 1/programmed death‐ligand 1 (PD‐1/PD‐L1) inhibitors in advanced esophageal squamous cell carcinoma (ESCC), their role in neoadjuvant therapy and reliable efficacy biomarkers remain elusive.

**Materials and Methods:**

We retrospectively analyzed locally advanced ESCC patients who underwent surgery following a 2‐cycle platinum and paclitaxel‐based treatment, with or without PD‐1 inhibitors (January 2020–March 2023). We assessed peripheral blood indexes and tertiary lymphoid structures (TLS) density to evaluate their impact on pathological response and prognosis, leading to a clinical prediction model for treatment efficacy and survival.

**Results:**

Of the 157 patients recruited, 106 received immunochemotherapy (ICT) and 51 received chemotherapy (CT) alone. The ICT group demonstrated a superior pathological response rate (PRR) (47.2% vs. 29.4%, *p* = 0.034) with comparable adverse events and postoperative complications. The ICT group also showed a median disease‐free survival (DFS) of 39.8 months, unattained by the CT group. The 1‐year DFS and overall survival (OS) rates were 73% and 91% for the ICT group, and 68% and 81% for the CT group, respectively.

We found higher baseline activated T cells, lower baseline Treg cells, and a decreased posttreatment total lymphocyte and CD4^+^/CD8^+^ ratio predicted an enhanced PRR. Reduced posttreatment CD4^+^/CD8^+^ ratio and increased NK cells were associated with prolonged survival, while higher TLS density indicated poorer prognosis. Among ICT group, a lower posttreatment CD4^+^/CD8^+^ ratio indicated longer DFS and reduced posttreatment B cells indicated longer OS. A nomogram integrating these predictors was developed to forecast treatment efficacy and survival.

**Conclusion:**

The combination of PD‐1 inhibitors and chemotherapy appears promising for locally advanced ESCC. Evaluating the differentiation status and dynamic changes of peripheral blood immune cells may provide valuable predictive insights into treatment efficacy and prognosis.

## 1 INTRODUCTION

Esophageal cancer (EC) ranks ninth in global cancer prevalence and stands as the sixth leading cause of cancer‐related mortality. Esophageal squamous cell carcinoma (ESCC) is the predominant pathological type of EC in China.^1^ Despite advances in therapeutic strategies extending survival, the significant burden of ESCC remains unmitigated.

Immunotherapy, notably immune checkpoint blockers (ICB), has captured considerable attention since its introduction. Numerous clinical trials have demonstrated that immunotherapy offers definitive efficacy and long‐term benefits compared to chemotherapy for advanced ESCC.^2^, ^3^ However, the discussion regarding its perioperative application, particularly in neoadjuvant therapy, remains ongoing.^4^ Pinpointing individuals who derive most benefit from immunotherapy remains a formidable challenge. Programmed death‐ligand 1 (PD‐L1) expression and tumor mutation burden have been sanctioned as biomarkers by the Food and Drug Administration and are widely utilized. However, their predictive capacity is constrained in patients with negative PD‐L1 expression or levels below 50%.^5^, ^6^

Cancer‐related inflammation is recognized as the seventh hallmark of cancer, and its intricate relationship with tumors operates as a double‐edged sword.^7^ Emerging evidence suggests a correlation between systemic inflammatory response, nutritional status and tumorigenesis.^8^ Principal inflammation‐based prognostic scores include a neutrophil‐to‐lymphocyte ratio (NLR), platelet‐to‐lymphocyte ratio (PLR), lymphocyte‐to‐monocyte ratio (LMR), and Systemic immune inflammation (SII).^9^, ^10^ Typical nutrition‐based prognostic scores are prognostic nutritional index (PNI), a critical indicator of nutritional and immune status.^11^, ^12^, ^13^ Immune function is carried out by immune cells, including T lymphocytes, B lymphocytes, and NK cells, are integral to tumor‐associated inflammation and exhibit pivotal anti‐tumor activity, which has increasingly gained attention.^9^, ^14^, ^15^, ^16^ These lymphocytes can be classified into peripheral blood lymphocytes and tumor‐infiltrating lymphocytes (TILs). The former, upon cytokine activation, differentiate into various subsets performing functions such as antigen presentation and tumor cell eradication. They migrate to tumor tissue in response to specific signals, forming TILs that modulate immune activation or escape.^17^ TILs, associated with the tumor microenvironment, infiltrate tumors to form tertiary lymphoid structures (TLS), which are differentiated lymphocyte clusters with lymph node‐like characteristics in response to stimuli. The presence of TLS in most tumors is believed to orchestrate a localized and sustained immune response.^18^, ^19^

In this study, we compiled data from 157 patients who underwent neoadjuvant therapy and surgery. Based on the pathological response results, patients were divided into two groups: a postoperative pathological response group (pPR, including pCR and MPR) and a non‐pathological response group (nPR). We investigated the correlation between peripheral blood inflammatory markers, immune‐related lymphocytes, and the pathological response rate (PRR) and patient survival. Moreover, we examined the relationship between relevant indicators and efficacy and prognosis in patients undergoing neoadjuvant immunotherapy, with the goal of predicting treatment outcomes more accurately.

## 2 MATERIALS AND METHODS

### 2.1 Study design

We conducted a retrospective analysis of patients with locally advanced ESCC, devoid of distant metastasis, who received chemotherapy with or without the PD‐1 inhibitor at the Affiliated Tumor Hospital of Nanjing Medical University. The inclusion criteria encompassed: confirmed diagnosis of locally advanced ESCC with at least one measurable lesion (according to Response Evaluation Criteria in Solid Tumors, RECIST1.1); no prior anti‐PD‐1/PD‐L1 treatment; after a 2‐cycle neoadjuvant treatment regime (platinum‐based chemotherapy combined with paclitaxel, with or without PD‐1 inhibitor) then underwent surgical resection at our hospital while retaining tissue pathology specimens and availability of complete data on systemic inflammation parameters and peripheral blood lymphocytes flow cytometry data at baseline and posttreatment. Exclusion criteria included: (1) severe comorbidities such as congestive heart failure, renal and/or hepatic failure, severe diabetes, etc.; (2) history of mental illness or other malignant tumors; (3) special populations such as pregnant and lactating women. (Please refer to Figure S1B for a detailed flowchart and inclusion and exclusion criteria). A total of 157 patients from January 2020 to March 2023 were included, with the last follow‐up date on April 19, 2023. All patients underwent surgical resection after 2 cycles of neoadjuvant treatment. Regular follow‐up were conducted, with an examination every 2–3 months using computed tomography imaging within the first year and performed every 6 months until disease recurrence/metastasis or death (showed in Figure S1A). This study was approved by the Ethics Committee of Jiangsu Cancer Hospital.

### 2.2 Clinical characteristics and laboratory parameters

Clinical characteristics and laboratory parameters for each patient were procured from the electronic medical records. Clinical characteristics included age, gender, Eastern Cooperative Oncology Group performance status (ECOG PS), tumor location and length, cancer staging, differentiation, 2‐cycle effect, pathological response, recurrence, and survival time. Laboratory parameters included carcinoembryonic antigen (CEA), peripheral blood inflammatory markers including NLR, PLR, LMR, SII, and PNI, calculation methods showed in Table S1. Peripheral blood lymphocyte subsets were sorted by flow cytometry based on surface markers, including total lymphocytes, CD4^+^ T cells, CD8^+^ T cells, activated T cells, regulatory T cells (Treg), B cells, natural killer cells (NK cells), and NKT‐like cells, antibody panels showed in Table S2. Data was collected at baseline and post 2‐cycle treatment to conduct an analysis of these indicators and assessed in relation to patients' pathological response and prognosis.

Pathological evaluation post‐surgery referred to the criterion of the College of American Pathologists/National Comprehensive Cancer Network (NCCN): All enrolled patients' hematoxylin and eosin (H&E) slides were graded as 0 (pCR, no evidence of vital residual tumor cells), 1 (MPR, 10% or less vital residual tumor cells), 2 (residual cancer foci with interstitial fibrosis), and 3 (few or no tumor cell regression) under the microscope by pathologists, with grades 0 and 1 defined as pCR while grades 2 and 3 as nPR. Survival was evaluated by disease‐free survival (DFS, time from surgical resection to recurrence/metastasis) and overall survival (OS, time from surgical resection to last follow‐up/death).

### 2.3 Tertiary lymphoid structures and calculation of density

The number of dense lymphocytic aggregates was quantified per 10× high‐power field in all tumor containing H&E stained diagnostic sections. TLS density had been calculated as the number of TLS per mm^2^ in tumoral regions. Patients considered as germinal centers positive (GC‐positive) if at least one TLS showed the characteristic morphology of proliferating centroblasts. All pathology sections are evaluated by two pathologists individually and blindly of the clinical data.

The TLS density was calculated in intertumoral and peritumoral 5 mm locations using an Olympus microscope BX51 marked with an eyepiece with a field of view of 22. TLS were counted per 10× field. Diameter (*d*) = 0.22 mm, S = p1/4*d* ^2^ = 3.8 mm^2^. TLS density = Total of 5 random views/5/S, as described previously.^20^

### 2.4 Statistical analyses

Statistical analyses were performed using RStudio (version 4.1.2) and SPSS (version 26.0). Best cut‐off of CEA, NLR, PLR, LMR, SII, PNI, and lymphocyte subsets were determined using the receiver operating characteristics (ROC) curve analysis, the best cut‐off was the one that maximized the Area under the curve (AUC). Comparative analysis between pPR and nPR groups was facilitated using the Mann–Whitney *U* test, chi‐square, or Fisher's exact test, while the paired *t*‐test was employed before and after treatment. Preliminary univariate logistic regression analysis was conducted on the variables, followed by the integration of those with *p* < 0.1 into the multivariate regression analysis. Survival analysis was executed employing the Kaplan–Meier method and the log‐rank test. The Cox proportional hazards regression analysis was utilized to discern potential indicators correlated with DFS and OS. Elements with *p* < 0.1 in the univariate analysis were incorporated into a multivariate analysis to identify independent prognostic factors. Nomograms for each predictive factor were constructed to forecast survival at 365‐ and 730‐days. Each nomogram was further validated internally using a bootstrap method with 1000 resamples. The discriminative power of the model was evaluated using the AUC value and the concordance index (C‐index), while calibration curves were employed to assess the correlation between actual outcomes and predicted probabilities.

## 3 RESULTS

### 3.1 Efficacy of neo‐immunochemotherapy in locally advanced ESCC

In this investigation, a cohort of 157 patients with advanced ESCC was recruited, among which 106 participants underwent immunochemotherapy (ICT), while the remaining 51 received neoadjuvant therapy consisting of platinum and paclitaxel chemotherapy (CT). Following the respective treatments, all patients underwent R0 resection surgery within a period of 30–40 days. In the ICT group, 33 patients (31.1%) achieved pCR, 17 patients (16.0%) reached MPR. In contrast, in the CT group, 7 patients (13.7%) attained pCR, and 8 patients (15.7%) reached MPR. The pathologic response rate (PRR, including pCR and MPR) were 47.2% in ICT and 29.4% in CT (*p* = 0.034). The incidence of grade 3–4 treatment‐related adverse events (AEs) was 44.3% in ICT and 31.4% in CT (*p* = 0.121) and surgical complications was 28.3% in ICT and 23.5% in CT (*p* = 0.527). No treatment‐related deaths were observed, indicating good tolerability. Detailed information on the baseline characteristics of the patients can be found in Table S3.

**FIGURE 1:**
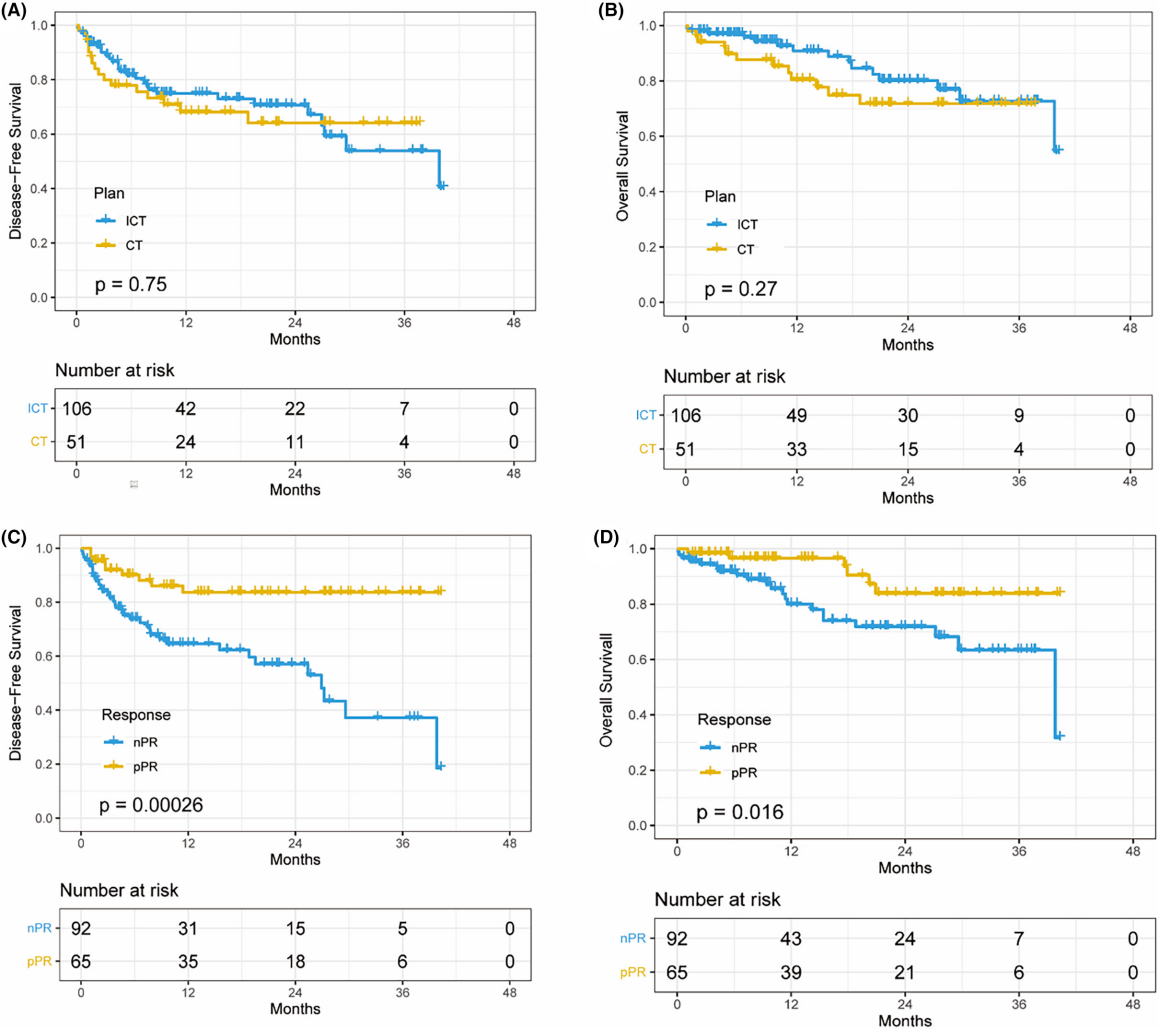
Kaplan–Meier curves and log‐rank test. (A, B) Kaplan–Meier curves of DFS and OS for neoadjuvant therapy regimen. (C, D) Kaplan–Meier curves of DFS and OS for pathological response.

The final follow‐up was conducted on April 19, 2023, the median follow‐up time was 19.5 months (95% Confidence interval, (CI): 14.0–25.0) from the first day of surgical resection and 2 patients lost follow‐up. Recurrence was observed in 29 patients (27.4%) in ICT and 16 patients (31.4%) in CT (*p* = 0.602). The median DFS in ICT was 39.8 months (95% CI: 22.4‐NA) and in CT was not reached (Figure 1A). The median OS in ICT group and CT group was not reached (Figure 1B). There were no significant differences observed between the two groups (*p* = 0.750 for DFS, *p* = 0.270 for OS). 1‐year DFS of 73% and OS of 91%, and a 2‐year DFS of 53% and OS of 80% in ICT and 1‐year DFS of 68% and OS of 81%, and a 2‐year DFS of 64%, and OS of 72% in CT.

In the nPR group, the median DFS time was 26.9 months (95%CI: 18.2–35.6), and the median OS was 39.8 months (95%CI: 25.5‐NA). The pPR group did not reach the median DFS and OS (Figure 1C,D) and showed significantly prolonged DFS (*χ* ^2^ = 13.359, *p* < 0.001, Figure 1C) and OS compared to nPR (*χ* ^2^ = 5.781, *p* = 0.016, Figure 1D and Table 1).

**TABLE 1:** Patients' baseline characteristics on nPR group and pPR group. ECOG PS, Eastern Cooperative Oncology Group Performance Status.

Parameters	nPR (%)	pPR (%)	*p*
Plan			
ICT	56 (60.87)	50 (76.92)	0.034
CT	36 (39.13)	15 (23.08)	
Sex			
Male	75 (81.52)	52 (80.00)	0.811
Female	17 (18.48)	13 (20.00)	
Age			
<66	55 (59.78)	33 (50.77)	0.262
≥66	37 (40.22)	32 (49.23)	
ECOG PS			
0	69 (75.00)	47 (72.31)	0.913
1	18 (19.57)	15 (23.08)	
2	5 (5.43)	3 (4.62)	
Preoperative radiotherapy			
No	84 (91.30)	48 (73.85)	0.003
Yes	8 (8.70)	17 (26.15)	
Tumor location			
Low	38 (41.30)	28 (43.08)	0.906
Middle	48 (52.17)	32 (49.23)	
Upper	6 (6.52)	5 (7.69)	
Tumor length^a^	2.50 (2.00–3.50)	2.20 (1.50–3.00)	0.016
Smoking			
No	83 (90.22)	59 (90.77)	0.908
Yes	9 (9.78)	6 (9.23)	
Drinking			
No	84 (91.30)	60 (92.31)	0.822
Yes	8 (8.70)	5 (7.69)	
Adverse event			
No	58 (63.04)	36 (55.38)	0.335
Yes	34 (36.96)	29 (44.62)	
Effect			
Partial response	32 (34.78)	34 (52.31)	0.044
Stable disease	58 (63.04)	31 (47.69)	
Progression disease	2 (2.17)	0 (0.00)	
Clinical nodal stage			
N0	7 (7.61)	0 (0.00)	<0.001
N1	31 (33.70)	43 (66.15)	
N2	49 (53.26)	22 (33.85)	
N3	5 (5.43)	0 (0.00)	
Downstaging of N stage			
No	39 (42.39)	5 (7.69)	<0.001
Yes	53 (57.61)	60 (92.31)	
Surgical complications			
No	67 (72.83)	48 (73.85)	0.887
Yes	25 (27.17)	17 (26.15)	
Differentiation			
I	16 (17.39)	9 (14.75)	<0.001
II	49 (53.26)	27 (44.26)	
III	26 (28.26)	13 (21.31)	
Unknown	1 (1.09)	12 (19.67)	

^a^ Means median and interquartile range (IQR).

### 3.2 The impact of periphery blood systemic inflammation parameters on efficacy and prognosis

**FIGURE 2:**
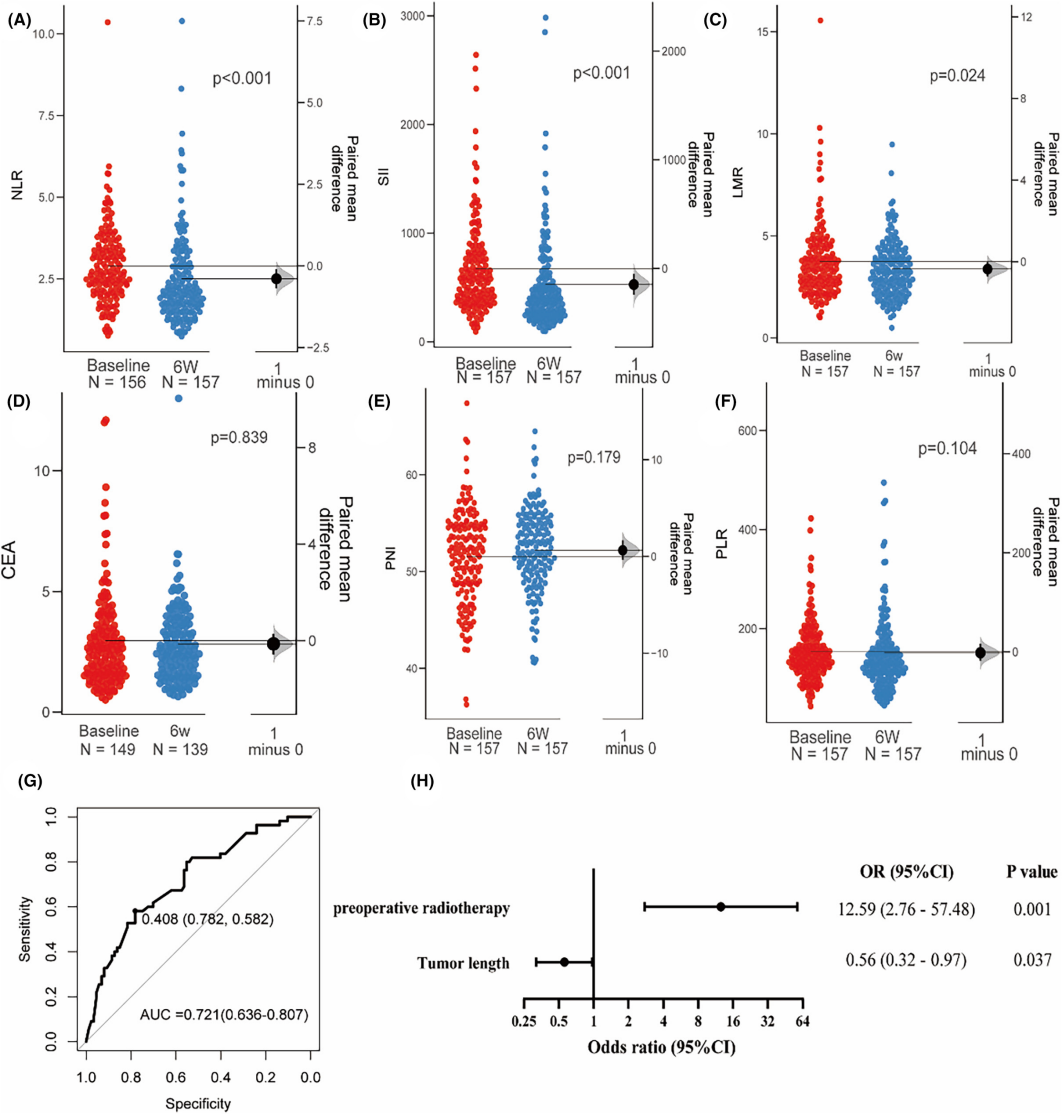
(A–F) Dynamic changes before and after neoadjuvant treatment in inflammatory markers, which show inflammatory markers on the left axes and paired mean difference is represented by the black dot on the right axes, along with the distribution (shaded curve) and 95% CI (black bars). (G) receiver operating characteristics curves and AUC value of the Logistic model about inflammatory markers. (H) Odds ratio and 95% CI of Logistic model about inflammatory markers showed in the Forest plot using a log2 scale on the x‐axis for better observation of difference.

We stratified patients into pPR and nPR cohorts and initially investigated the association between inflammatory markers and PRR and prognosis, the detailed characteristics are showed in Table S4. Our results indicated that there was a significant decrease in NLR, SII levels, and LMR (*p* < 0.001, *p* < 0.001, *p* = 0.024) following 2 cycles of treatment, (Figure 2A–C). However, no significant differential was discerned between the pPR and nPR groups (Figure S2). We incorporated these variables into univariate and multivariate logistic regression analyses (Table S5). The data suggested that preoperative radiotherapy (*p* = 0.001, OR = 12.59, 95% CI: 2.76–57.48) and tumor length (*p* = 0.037, OR = 0.56, 95% CI: 0.32–0.97) were significantly correlated with PRR. The AUC value of the logistic model was 0.721 (95% CI: 0.636–0.807, Figure 2G).

**FIGURE 3:**
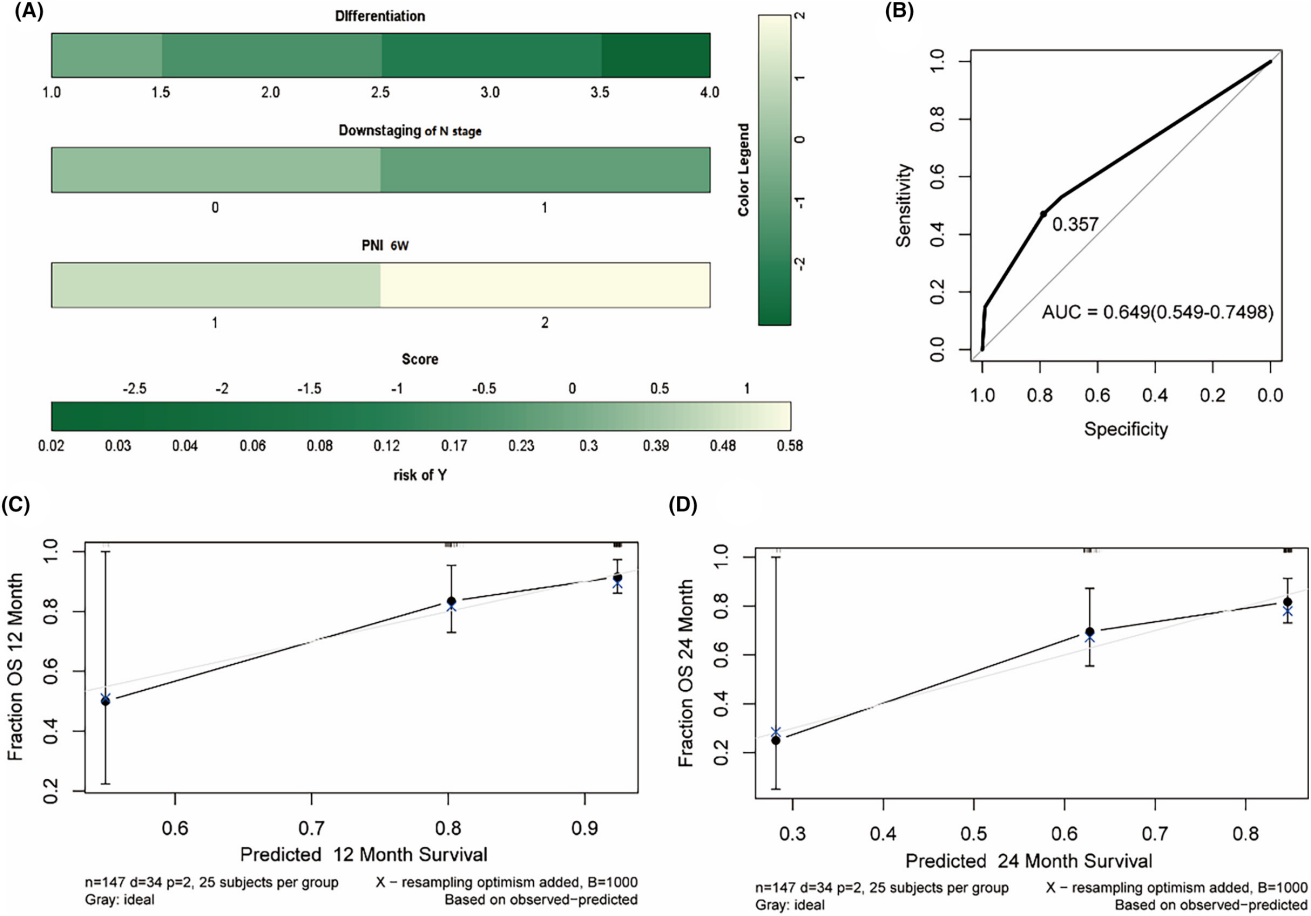
Analysis of therapeutic efficacy based on peripheral blood inflammatory markers. (A) Nomogram based on the multivariate model for OS. (B) ROC curves of the model. (C, D) The 365‐ and 730‐ days OS calibration curves of the nomogram.

The parameters were incorporated into a Cox regression. An elevated PNI post‐treatment was positively associated with DFS (*p* = 0.035, HR = 0.500, 95% CI: 0.260–0.950, Table S6), but negatively with OS (*p* = 0.043, HR = 2.480, 95% CI: 1.030–5.970, Table S7). Furthermore, lymph node downstaging (*p* = 0.037, HR = 0.420, 95% CI: 0.190–0.950) and differentiation (*p* = 0.032, HR = 0.530, 95% CI: 0.290–0.950) were associated with a prolonged OS. To enhance predictive efficacy and clinical applicability, a nomogram for OS was developed (Figure 3D), yielding a C‐index of 0.812 and an AUC value of 0.700 (95% CI: 0.593–0.738, Figure 3E), indicating good discriminatory power and moderate accuracy in predicting OS outcomes. The 365‐ and 730‐day calibration curves exhibited correlations between actual and predicted outcomes (Figure 3F,G). The C‐index and AUC value of the DFS nomogram model were 0.734 and 0.598 (95% CI: 0.444–0.769), respectively (Figure S3), indicating moderate discriminative ability but poor accuracy. Additional consideration should be given to the wide confidence interval, indicating uncertainty in the AUC estimate.

### 3.3 The impact of lymphocyte subsets and TLS on PRR and prognosis

**FIGURE 4:**
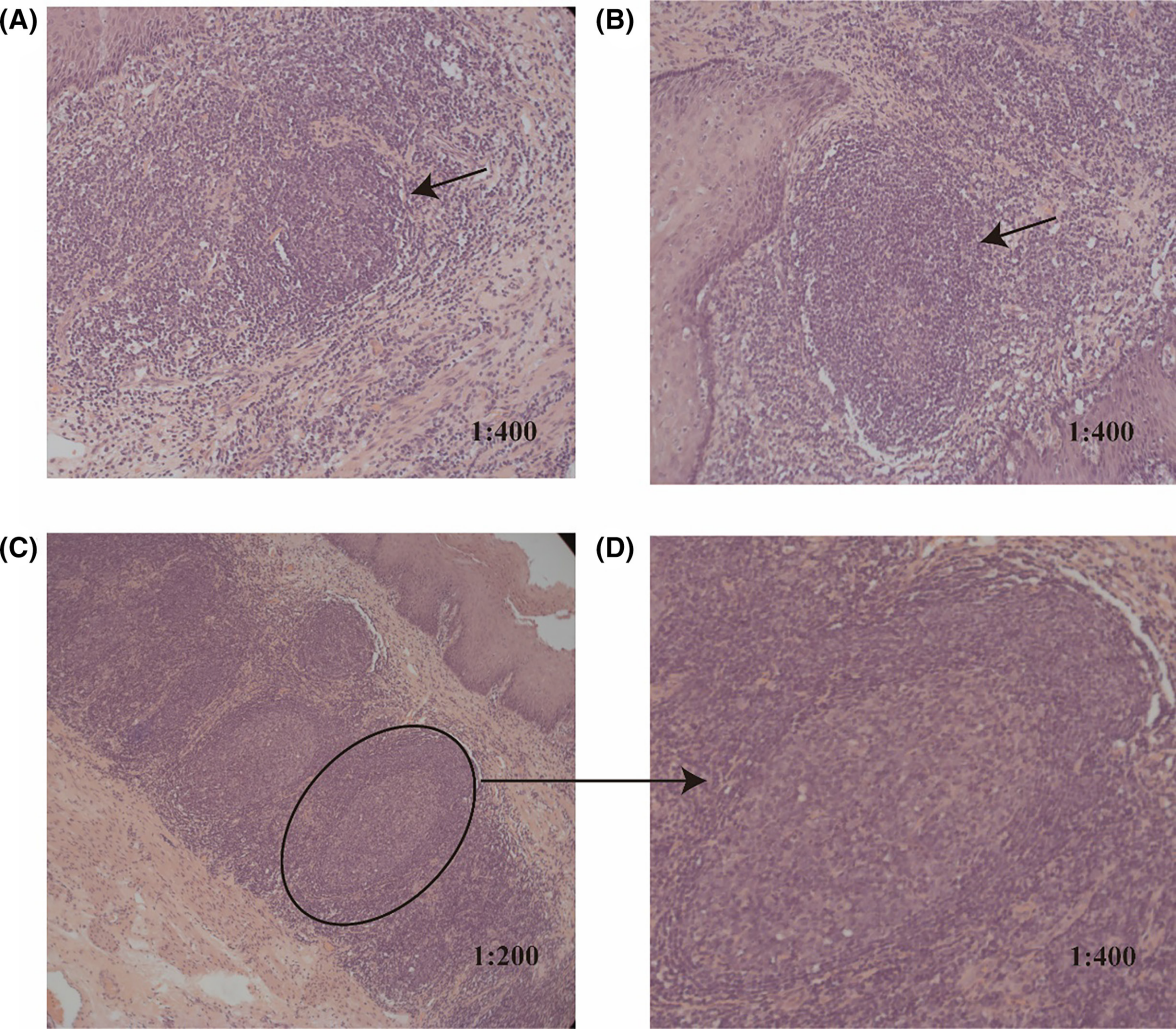
The tertiary lymphoid structures (TLS) in tumor tissue H&E sections. (A) Early TLS for lymphocyte aggregation. (B) Primary lymphoid structures. (C, D) Secondary lymphoid structures, germinal centers positive (GC‐positive).

Subsequently, we collected peripheral blood lymphocyte subsets pre‐ and post‐therapy and the density of TLS in 86 patients (Table S8). We investigated all pathological tissue sections and identified the presence of various stages of TLS within the tumor tissues (Figure 4A–D). To compute the density of TLS, we randomly selected five bright‐field fields under a microscope, and the TLS density was calculated as the number of TLS per mm^2^ in peritumoral regions.

**FIGURE 5:**
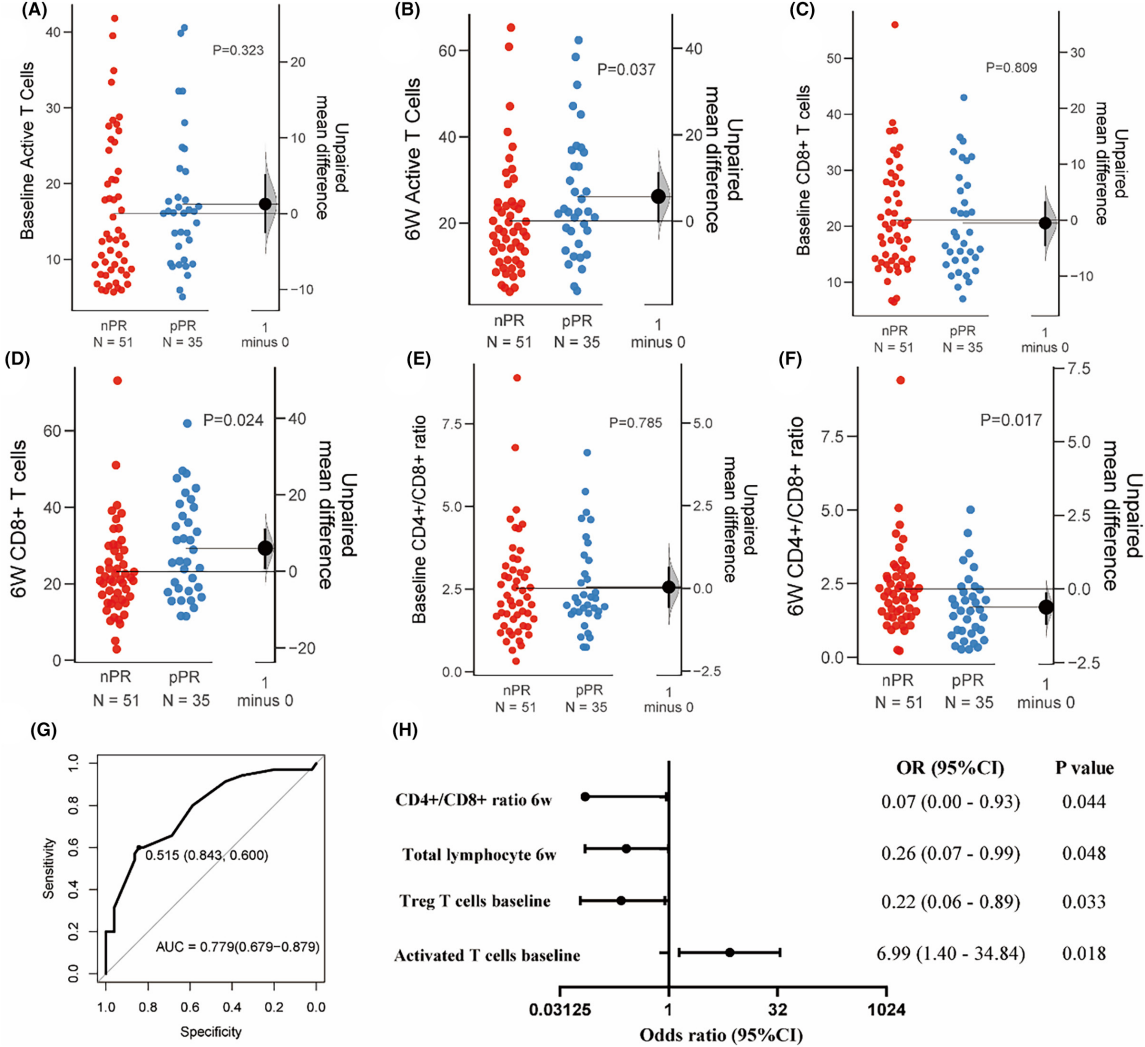
(A–F) show dynamic changes in lymphocyte subgroups between the pPR and nPR groups at baseline and after treatment, which show cell frequencies on the left axes and mean difference is represented by the black dot on the right axes, along with the distribution (shaded curve) and 95% CI (black bars). (G) receiver operating characteristics curves of the Logistic model about peripheral blood lymphocyte subsets. (H) Forest plot displaying the OR and 95% CI of the Logistic model.

Following 2 cycles of therapy, we detected a dynamic increase in CD8^+^ T cell (*p* = 0.024) and activated T cell (*p* = 0.037) in the pPR group (Figure 5A–D), accompanied by a decrease in the CD4^+^/CD8^+^ ratio (*p* = 0.017, Figure 5E,F). The density of TLS did not significantly differ between the two groups. Detailed information on the differences in lymphocyte subsets between the pPR group and nPR group refer to Figure S4, and changes before and after treatment refer to Figure S5.

We incorporated these variables into a Logistic regression model (Table S9) and found that elevated activated T cells at baseline were correlated with a higher PRR (*p* = 0.018, OR = 6.990, 95% CI: 1.400–34.840). Conversely, higher Treg cells at baseline (*p* = 0.033, OR = 0.220, 95% CI: 0.060–0.890), total lymphocyte count (*p* = 0.048, OR = 0.260, 95% CI: 0.070–0.990), and CD4^+^/CD8^+^ ratio posttreatment (*p* = 0.044, OR = 0.070, 95% CI: 0.000–0.930) indicated a lower PRR (Table S9). The model exhibited a good predictive capability, with an AUC of 0.779 (95% CI: 0.679–0.879, Figure 5K), which indicates reasonably good performance and suggests the model's effectiveness in predicting efficacy.

We subsequently explored the association between these parameters and prognosis. A heightened CD4^+^/CD8^+^ ratio was linked to a shorter DFS (*p* = 0.026, HR = 2.4, 95% CI: 1.11–5.17) while NK cells was associated with a longer DFS and OS (*p* = 0.028, HR = 0.360, 95% CI: 0.140–0.900; *p* = 0.040, HR = 0.210, 95% CI: 0.050–0.940, Tables S10 and S11). The density of TLS exhibited correlation with OS: higher levels indicative of a poorer OS (*p* = 0.030, HR = 1.250, 95% CI: 1.020–1.530).

**FIGURE 6:**
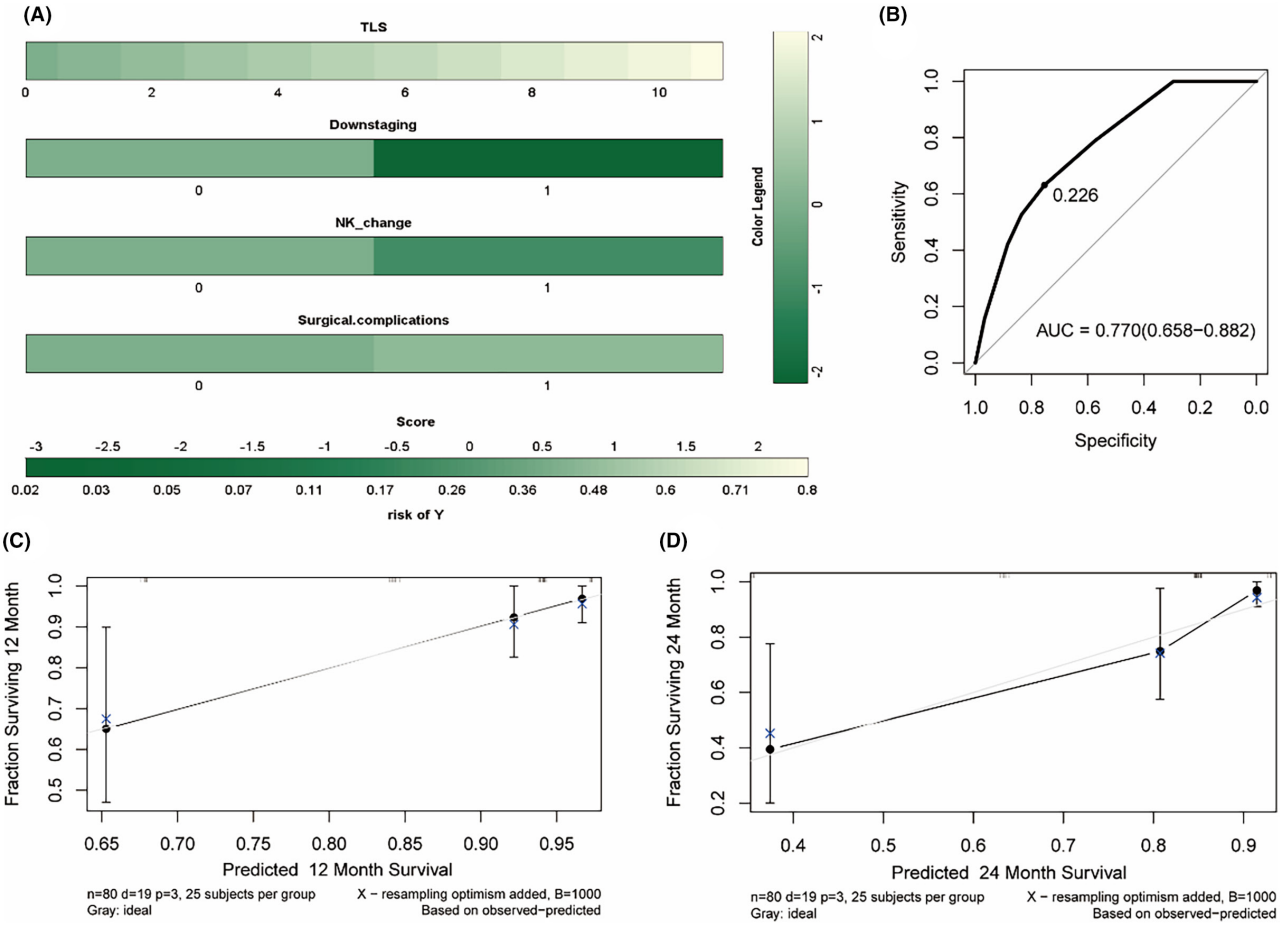
Analysis of prognosis based on peripheral lymphocyte subsets. (A) Nomogram based on the COX model in peripheral lymphocyte subsets. (B) receiver operating characteristics curves in OS nomogram. (C, D) The 365‐ and 730‐ days OS calibration curves.

Based on these significant predictors of OS, nomogram was constructed. The C‐index and AUC value of the OS nomogram model were 0.802 and 0.629 (95% CI: 0.592–0.667, Figure 6A). The 365‐ and 730‐day calibration curves displayed a notable correlation between actual and predicted outcomes. We also constructed predicted model of DFS, the C‐index and AUC value were 0.758 and 0.787 (95% CI: 0.563–0.933), respectively (Figure S6), exhibiting good discriminative ability and accuracy.

### 3.4 The prognostic value of inflammatory markers and lymphocyte subsets in the neoadjuvant immunochemotherapy cohort

Based on our findings, we discerned that ICT can enhance the PRR in locally advanced ESCC. Consequently, we further explore the predictive indicators of pathological response and prognosis within the ICT group.

**FIGURE 7:**
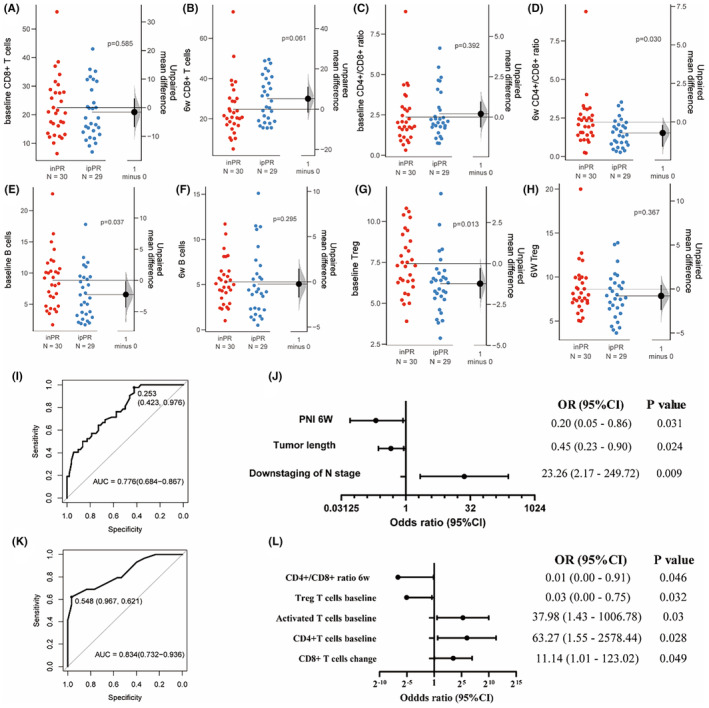
(A–H) Dynamic alterations about peripheral blood lymphocyte subsets in inPR and ipPR group, which show cell frequencies on the left axes and mean difference is represented by the black dot on the right axes, along with the distribution (shaded curve) and 95% CI (black bars). (I, J) receiver operating characteristics (ROC) curve and forest plot of the Logistic regression model concerning inflammatory markers. (K, L) ROC curve and forest plot of the Logistic regression model pertaining lymphocyte subgroups.

We segregated the 106 patients who received neoadjuvant immunotherapy into ipPR and inPR groups and analyzing the correlation between the immune‐related features above and efficacy as well as prognosis. After 2 cycles of immune therapy, the levels of CD3^+^ T cells (*p* = 0.019), CD8^+^ cells (*p* = 0.003), activated T cells (*p* < 0.001), and Treg cells (*p* < 0.001) increased, while the levels of the CD4^+^/CD8^+^ ratio (*p* = 0.008) and B cells (*p* < 0.001) decreased (Figure S9). Furthermore, when comparing the ipPR and inPR groups, we found that CD8^+^ T cells showed a significant increase after treatment, accompanied by a decrease in the CD4^+^/CD8^+^ ratio. Additionally, B cells and Treg cells, which had initially been low at baseline, exhibited an increase after treatment (Figure 7A–H).

We discovered that an increase in PNI levels after treatment signified a poorer PRR (*p* = 0.031, OR = 0.200, 95% CI: 0.050–0.860, Table S12). In alignment with the overall population, lower activated T cells (*p* = 0.030, OR = 37.980) and higher Treg cells levels (*p* = 0.032, OR = 0.030, 95% CI: 0.000–0.750) at baseline and higher level of CD4^+^/CD8^+^ ratio post‐therapy (*p* = 0.046, OR = 0.010, 95% CI: 0.000–0.910) indicated that patients had a suboptimal response to immunotherapy (Table S13). We constructed Logistic model of inflammatory markers and lymphocyte subsets in the ipPR group, The AUC was 0.776 (95% CI: 0.684–0.867, Figure 7I) and 0.834 (95% CI: 0.732–0.936, Figure 7K), respectively.

Subsequently, we constructed COX regression models and found that, in line with the overall population, a higher PNI posttreatment was associated with extended DFS and a lower risk of recurrence (*p* = 0.004, HR = 0.300, 95% CI: 0.130–0.690, Table S14). CD4^+^ T cells at baseline, NK cell levels and TLS did not exhibit significant predictive value for DFS and OS. However, CD4^+^/CD8^+^ ratio still held predictive value for DFS (*p* = 0.022, HR = 3.320, 95% CI: 1.190–9.260, Figure S8 and Table S16). Furthermore, a dynamic decrease in B cells posttreatment indicated an elongated OS (*p* = 0.027, HR = 27.770, 95% CI: 1.470–256.210, Table S17), the dynamic changes of B cells may serve as a unique predictive indicator for neoadjuvant immunotherapy.

## 4 DISCUSSION

Building upon promising results observed in immunotherapy in the neoadjuvant treatment for lung cancer,^21^ our study aimed to explore its potential application in the neoadjuvant management of ESCC. Presently, numerous phase II clinical trials are in progress to evaluate the efficacy of an ICT as a neoadjuvant treatment in locally advanced ESCC, most showed promising pCR rates and manageable AEs.^22^ ChiCTR2100051903 study reports suggest the pCR rate was observed to be 31.3% with a tolerable AE rate.^23^ Zhang et al. reported promising rates of pathological response and manageable occurrence of AEs in ESCC receiving neoadjuvant ICT.^24^ Duan et al. studied pembrolizumab plus chemotherapy in ESCC, reporting acceptable toxicity and strong efficacy.^25^ In our study, with the incidence of AEs and surgical complications between ICT and CT comparable, ICT was found to increase PRR compared to CT, however, this rise did not result in a prolonged survival benefit. This suggests that neoadjuvant ICT may effectively control the primary site, but there may be residual small foci or other tiny metastatic sites, leading to recurrence or metastasis. Moreover, majority of studies primarily concentrate on efficacy without adequate clinical evidence demonstrating the survival benefits of neoadjuvant immunotherapy. Further investigation and research are essential.

The impact of systemic inflammatory response on various cancers is well‐established. Inflammatory markers are acknowledged as prognostic indicators for solid malignancies, including gastric cancer,^26^ colorectal cancer,^27^ lung cancer,^28^ and esophageal cancer.^10^ However, conflicting finding exist. Wang et al.^29^ and Qi et al.^30^ reported that certain inflammatory markers, including NLR, PLR, LMR, SII, and PNI, did not predict efficacy in ESCC undergoing different therapies. In our study, while NLR, PLR, LMR, and SII did not correlate with ICBs efficacy, posttreatment PNI was associated with survival outcomes. Elevated PNI signified a longer DFS in both the overall and ICT group but correlated with poorer OS in the overall population. This suggests an increase in PNI may reflect a favorable immune system status, contributing preventing or delaying tumor spread, but it may lead to more unfavorable survival outcomes by influencing tumor growth and systemic metabolic processes.^31^ Certain tumor subpopulations may exhibit immune evasion, resulting in the inability of the body to completely eradicate tumor cells and consequently leading to a reduction in overall survival.^7^, ^11^ Therefore, when evaluating the prognosis of immunotherapy patients, it is essential to comprehensively consider various factors and conduct a comprehensive analysis by integrating clinical and experimental data.

Immunotherapy aims to activate or enhance immune system to identify, attack, and eliminate tumor cells by activating immune lymphocytes, driving the differentiation of lymphocyte subsets, and exerting anti‐tumor effects.^32^ T lymphocytes are at the core of the immune response and can be functionally categorized into subtypes including Helper T cells (Th), Suppressor T cells (Ts), Effector T cells (Te), and Cytotoxic T cells (CTL). Among these subtypes, Te and Ts are closely associated with treatment response.^33^ Te cells, also referred to as activated T cells, have been associated with better responses to immunotherapy in previous studies. Our study demonstrated that higher baseline activated T cells predict better PRR, with similar results observed in the ICT group, suggesting that the level of activated T cells may reflect the body's immune status and anti‐tumor ability. Monitoring the levels of activated T cells in peripheral blood during treatment may help evaluate efficacy. However, activated T cells were marked by HLADR, a general marker for various activated T lymphocytes, and failed to further distinguish between effector T cells and exhausted T cells to differentiate their immune functional states.^34^ Future research should focus on further delineating lymphocyte subsets to elucidate the relationship between these cell types and treatment efficacy as well as prognosis. Treg, belonging to the Ts subtype, exerts potent immunosuppressive effects in the tumor microenvironment.^35^ Various studies have indicated that an increase in infiltrating Treg cells is linked to immune suppression and tumor progression.^36^, ^37^ One study suggested that patients with a higher proportion of CD4^+^CD25^+^ Treg cells was associated with a poorer prognosis in gastric cancer.^38^ Consistent with prior research, our study observed a negative correlation between baseline Treg levels and pathological response. This findings complement existing viewpoints and highlight the importance of Treg cells in immunotherapy.

In addition to the cell types discussed above, CD8^+^ T lymphocytes also play a crucial role in the immune environment as they can differentiate into CTLs that directly target and kill tumors. Previous studies have highlighted that higher levels of CD8^+^ T cells, CD4^+^ T cells, and PD‐L1 in tumor tissues are associated with a favorable response to immunotherapy.^32^, ^39^ In this study, following 2 cycles of treatment, the pPR group exhibited a decrease in CD4^+^ T cells and an increase in CD8^+^ T cells, leading to a significant decrease in the CD4^+^/CD8^+^ ratio, which was associated with better efficacy and prognosis. Consistent with previous research, we observed the same results in the ICT group. We hypothesize that posttreatment activation of immune system, reduction of immune suppressive factors in the tumor microenvironment, contribute to proliferation and activation of CD8^+^ T lymphocytes, thus correlating with a favorable cytotoxic effects and treatment response. Therefore, peripheral blood lymphocyte subsets can reflect immune status and provide important reference value for assessing treatment responsiveness and prognosis.

This study observed a significant increase in TILs within tumor tissues post‐neoadjuvant therapy, with the development of TLS in some patients. Comprising follicular B cells, dendritic cells (DCs), T cells, fibroblasts, and other components, TLS form immune cell aggregates resembling lymph node characteristics.^40^ The predominant localization of TLS in the submucosa of esophageal squamous epithelium, nearly absent in the epithelial and muscular layers, may be attributed to the vascular‐rich environment facilitating immune cell recruitment. Analysis of enrolled patients revealed that higher TLS density was linked to inferior DFS. However, no predictive value was observed in the ICT group. The role of TLS remains highly debates. Cabrita et al.^41^ reported a positive association between TLS presence and improved immunotherapy response and survival rates in melanoma patients, while Firkin's team noted a correlation between TLS and poor prognosis in early hepatocellular carcinoma tissue.^42^ Existing studies predominantly focus on advanced cancer, with inconclusive findings regarding the predictive value of TLS in evaluating efficacy and prognosis in early‐stage tumors or neoadjuvant immunotherapy.^43^, ^44^ Variability and even contradictions in the association of TLS in different tumor regions and maturation stages with tumor recurrence and progression underscore the necessity for further research into the relationship of TLS with immunotherapy response and prognosis.

However, this study has certain limitations. Firstly, this retrospective clinical study involved a limited patient cohort with a relatively short follow‐up duration, limiting the ability to compare prognostic differences between cohorts. Secondly, the assessment of TLS is subjective and relies on pathologists evaluating them under a microscope. Despite efforts to minimize bias, there may still be inaccuracies that could impact the predictive capabilities of subsequent models. Thirdly, while we have developed a clinical prediction model, it has not undergone external validation or comparison with established clinical models. Future studies will focus on increasing sample size, extending follow‐up periods, and conducting comparative analyses with existing models.

Despite these limitations, our study offers valuable insights into neoadjuvant treatment strategies for locally advanced ESCC. It highlights the potential to assess immune status using peripheral blood and pathological tissue specimens to evaluate treatment efficacy and prognostic value. This approach offers a guiding method for early prognosis determination during clinical treatment, ultimately improving patient outcomes and survival. Moving forward, enhancing immunotherapy efficacy through targeted drug interventions to activate specific immune cell subpopulations holds promise for extending patient survival.

## AUTHOR CONTRIBUTIONS

**Ruotong Wang:** Conceptualization (equal); formal analysis (equal); investigation (equal); writing – original draft (equal). **Shaodi Wen:** Conceptualization (equal); formal analysis (equal); methodology (equal); writing – original draft (equal). **Xiaoyue Du:** Investigation (equal); writing – original draft (equal). **Jingwei Xia:** Investigation (equal); writing – original draft (equal). **Bowen Hu:** Investigation (equal); writing – original draft (equal). **Yihan Zhang:** Investigation (equal); writing – original draft (equal). **Guoren Zhou:** Resources (equal); writing – original draft (equal). **Feng Jiang:** Resources (equal); writing – original draft (equal). **Xiaomin Lu:** Resources (equal); writing – original draft (equal). **Miaolin Zhu:** Conceptualization (equal); resources (equal). **Xinyu Xu:** Conceptualization (equal); resources (equal). **Bo Shen:** Funding acquisition (equal); project administration (equal); resources (equal).

## FUNDING INFORMATION

This work was supported by Bo Shen: the National Natural Science Foundation of China (No.82272863); Hengrui Cancer Research Fund, Hengrui Pharmaceutical Clinical Research Fund, (No.Y‐HR2020MS‐0485, No.JZ21449020210607).

## CONFLICT OF INTEREST STATEMENT

The authors have declared that there is no conflict of interest.

## ETHICS STATEMENT

The study protocol was approved by the Ethics Committee of Jiangsu Cancer Hospital. The requirement for informed consent was waived by the Jiangsu Cancer hospital ethics committees due to the retrospective nature of the study. All data were anonymized. All methods in the study were carried out in accordance with relevant guidelines and regulations (declaration of Helsinki).

## Supporting information


Data S1.

## Data Availability

The data that support the findings of this study are available from the corresponding author upon reasonable request.
