# Supplementary material for: The efficacy of neoadjuvant immunotherapy and lymphocyte subset predictors in locally advanced esophageal squamous cell carcinoma: A retrospective study
Source: Cancer Med. 2024 Sep 14;13(17):e70228. doi: 10.1002/cam4.70228 (PMC11399715; doi:10.1002/cam4.70228)
Supplement: Supplementary file 1 — Data S1. [file CAM4-13-e70228-s001.docx]

# The Efficacy of Neoadjuvant Immunotherapy and Lymphocyte Subset Predictors in Locally Advanced Esophageal Squamous Cell Carcinoma: A Retrospective Study.

Ruotong Wang^1†^, Shaodi Wen^1†^, Xiaoyue Du^1^, Jingwei Xia^1^, Bowen Hu^1^, Yihan Zhang^1^, Guoren Zhou^1^, Feng Jiang^3^, Xiaomin Lu^4^, Miaolin Zhu^2*^, Xinyu Xu^2*^, Bo Shen^1,4,5*^

^†^Ruotong Wang and Shaodi Wen contributed equally to this work.

^1^Department of Oncology, The Affiliated Cancer Hospital of Nanjing Medical University, Jiangsu Cancer Hospital, Jiangsu Institute of Cancer Research, Nanjing 210009, China;

^2^Department of Pathology, The Affiliated Cancer Hospital of Nanjing Medical University, Jiangsu Cancer Hospital, Jiangsu Institute of Cancer Research, Nanjing, 210009, China;

^3^Department of Thoracic Surgery, The Affiliated Cancer Hospital of Nanjing Medical University, Jiangsu Cancer Hospital, Jiangsu Institute of Cancer Research, Nanjing 210009, China;

^4^Department of Oncology, Affiliated Haian Hospital of Nantong University, Haian, Nantong 226600, China; ^5^Department of Oncology, Huaian Hospital of Huaian City, Huaian Cancer Hospital, Huaian 223200, China.

**Correspondence to*:

Bo Shen. Department of Oncology, The Affiliated Cancer Hospital of Nanjing Medical University, Jiangsu Cancer Hospital, Jiangsu Institute of Cancer Research, Nanjing 210009, China. Email: [shenbo987@njmu.edu.cn](mailto:shenbo987@njmu.edu.cn);

Xinyu Xu. Department of Pathology, The Affiliated Cancer Hospital of Nanjing Medical University, Jiangsu Cancer Hospital, Jiangsu Institute of Cancer Research, Nanjing, 210009, China. Email: [xxyblk@163.com](mailto:xxyblk@163.com);

Miaolin Zhu. Department of Pathology, The Affiliated Cancer Hospital of Nanjing Medical University, Jiangsu Cancer Hospital, Jiangsu Institute of Cancer Research, Nanjing, 210009, China. Email: zhumiaolin829yyds@gmail.com.

**
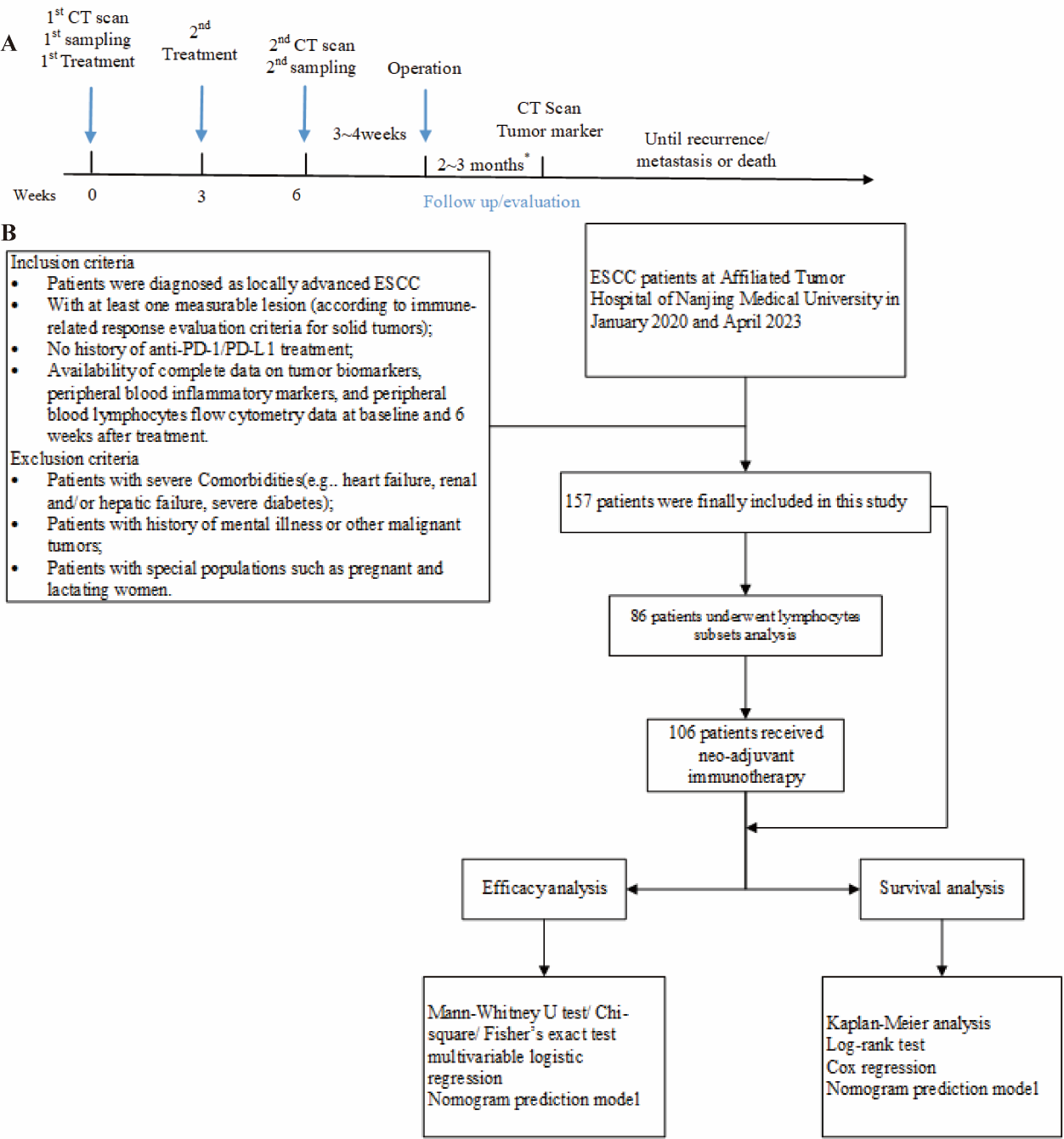
**

**Supplementary** **Figure 1 A** The flowchart of this study. ^*^Within the first year, a follow-up examination was conducted every 2-3 months using CT imaging. Subsequently, CT scans were performed every 6 months until disease recurrence/metastasis or death. **B** The detailed inclusion and exclusion criteria of this study.

**Supplementary Table 1**

| Periphery blood systemic inflammation | Calculation methods |
| --- | --- |
| NLR | absolute neutrophil count/absolute lymphocyte count |
| PLR | absolute platelet count/absolute lymphocyte count |
| LMR | absolute lymphocyte count/monocyte |
| SII | neutrophil count*platelet count/lymphocyte count |
| PNI | serum albumin+5×peripheral blood lymphocyte count |

**Supplementary Table 2** Flow cytometry gating strategy based on cell surface markers.

| Marker | Lymphocyte subsets |
| --- | --- |
| CD45^+^ | Total lymphocytes |
| CD45^+^CD3^+^ | Total T cells |
| CD45^+^CD3^+^CD4^+^ | CD4^+^ T cells |
| CD45^+^CD3^+^CD8^+^ | CD8^+^ T cells |
| CD45^+^CD3^+^HLADR^+^ | Activated T cells |
| CD4^+^CD25^+^CD127^-^ | Treg cells |
| CD45^+^CD3^-^CD19^+^ | B cells |
| CD45^+^CD3^-^CD56^+^ | NK cells |
| CD45^+^CD3^+^CD56^+^ | NKT-like cells |

**Supplementary Table 3** Patients’ baseline characteristics on ICT group and CT group.

| **Parameters** | **ICT n(%)** | **CT n(%)** | **P** |
| --- | --- | --- | --- |
| **Sex** |  |  |  |
| Male | 89(84.0) | 38(74.5) | 0.158 |
| Female | 17(16.0) | 13(25.5) |  |
| **Age** |  |  |  |
| <66 | 62(58.5) | 26(51.0) | 0.375 |
| ≥66 | 44(41.5) | 25(49.0) |  |
| **ECOG PS** |  |  |  |
| 0 | 80(75.5) | 36(70.6) | 0.600 |
| 1 | 20(18.9) | 13(25.5) |  |
| 2 | 6(5.7) | 2(3.9) |  |
| **Smoking** |  |  |  |
| Y | 11(10.4) | 4(7.8) | 0.829 |
| N | 98(89.6) | 47(92.2) |  |
| **Drinking** |  |  |  |
| Y | 8(7.5) | 5(9.8) | 0.864 |
| N | 98(92.5) | 46(91.7) |  |
| **Pathologic response** |  |  |  |
| pCR | 33(31.1) | 7(13.7) | 0.034 |
| MPR | 17(16.0) | 8(15.7) |  |
| other | 56(52.9) | 36(70.6) |  |
| **2-cycle Effect** | |  |  |
| PR | 47(44.3) | 19(37.3) | 0.637 |
| SD | 58(54.7) | 31(60.8) |  |
| PD | 1(0.9) | 1(2.0) |  |
| **Adverse event** |  |  |  |
| Y | 47(44.3) | 16(31.4) | 0.121 |
| N | 59(55.7) | 35(68.6) |  |
| **Tumor location** |  |  |  |
| Upper | 47(44.3) | 19(37.3) | 0.293 |
| Middle | 50(47.2) | 30(58.8) |  |
| Low | 9(8.5) | 2(3.9) |  |
| **Tumor length**^†^ | 2.50(1.78-3.32) | 2.25(1.82-3.00) | 0.579 |
| **Clinical nodal stage** |  |  |  |
| N0/1 | 49(46.2) | 33(62.7) | 0.019 |
| N2 | 52(49.1) | 19(37.3) |  |
| N3 | 5(4.7) | 0(0) |  |
| **Downstaging of N**  **stage** |  |  |  |
| Y | 84(79.2) | 29(56.9) | 0.003 |
| N | 22(20.8) | 22(43.1) |  |
| **Differentiation** |  |  |  |
| Ⅰ | 14(13.7) | 11(21.6) | 0.059 |
| Ⅱ | 53(52.0) | 23(45.1) |  |
| Ⅲ | 23(22.5) | 16(31.4) |  |
| unknown | 12(11.8) | 1(2.0) |  |
| **Surgical**  **complications** |  |  |  |
| Y | 30(28.3) | 12(23.5) | 0.527 |
| N | 76(71.7) | 39(76.5) |  |
| **preoperative**  **radiotherapy** |  |  |  |
| Y | 23(21.7) | 2(3.9) | 0.009 |
| N | 83(78.3) | 49(96.1) |  |
| **Recurrence** |  |  |  |
| Y | 29(27.4) | 16(31.4) | 0.602 |
| N | 77(72.6) | 35(68.6) |  |

ECOG PS, Eastern Cooperative Oncology Group Performance Status. ^†^means median and interquartile range (IQR)

**
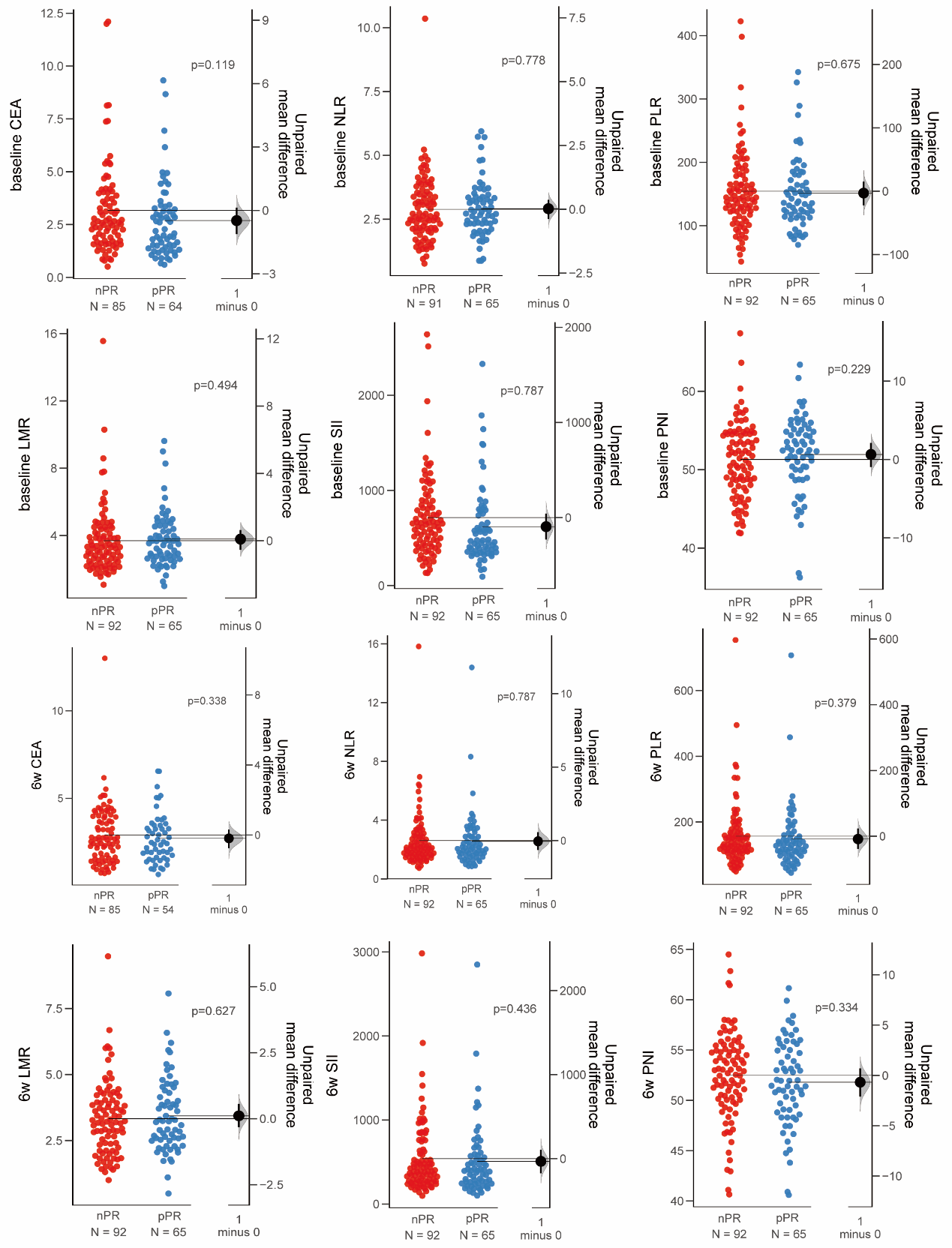
**

**Supplementary Figure 2** Dynamic changes about peripheral inflammatory markers between pPR group and nPR group.

**Supplementary Table 4** Patients’ baseline characteristics and periphery blood systemic inflammation parameters on nPR group and pPR group.

| **Parameters** | **nPR (%)** | **pPR (%)** | **P** |
| --- | --- | --- | --- |
| **CEA Baseline** |  |  |  |
| <1.56 | 13 (15.3) | 21 (32.8) | 0.020 |
| ≥1.56 | 72 (84.7) | 43 (67.2) |  |
| **NLR Baseline** |  |  |  |
| <2.27 | 30 (33.0) | 15 (23.1) | 0.244 |
| ≥2.27 | 61 (67.0) | 50 (76.9) |  |
| **PLR Baseline** |  |  |  |
| <126.32 | 28 (30.4) | 25 (38.5) | 0.381 |
| ≥126.32 | 64 (69.6) | 40 (61.5) |  |
| **LMR Baseline** |  |  |  |
| <3.31 | 48 (52.2) | 26 (40.0) | 0.179 |
| ≥3.31 | 44 (47.8) | 39 (60.0) |  |
| **SII Baseline** |  |  |  |
| <590.55 | 44 (47.8) | 39 (60.0) | 0.179 |
| ≥590.55 | 48 (52.2) | 26 (40.0) |  |
| **PNI Baseline** |  |  |  |
| <53.23 | 50 (54.3) | 43 (66.2) | 0.188 |
| ≥53.23 | 42 (45.7) | 22 (33.8) |  |
| **CEA 6W** |  |  |  |
| <2.16 | 24 (30.0) | 27 (45.8) | 0.084 |
| ≥2.16 | 56 (70.0) | 32 (54.2) |  |
| **NLR 6W** |  |  |  |
| <3.36 | 76 (82.6) | 52 (80.0) | 0.837 |
| ≥3.36 | 16 (17.4) | 13 (20.0) |  |
| **PLR 6W** |  |  |  |
| <147.45 | 52 (56.5) | 45 (69.2) | 0.148 |
| ≥147.45 | 40 (43.5) | 20 (30.8) |  |
| **LMR 6W** |  |  |  |
| <2.14 | 22 (23.9) | 8 (12.3) | 0.106 |
| ≥2.14 | 70 (76.1) | 57 (87.7) |  |
| **SII 6W** |  |  |  |
| <265.68 | 20 (21.7) | 20 (30.8) | 0.274 |
| ≥265.68 | 72 (78.3) | 45 (69.2) |  |
| **PNI 6W** |  |  |  |
| <51.48 | 34 (37.0) | 32 (49.2) | 0.171 |
| ≥51.48 | 58 (63.0) | 33 (50.8) |  |
| **CEA change** |  |  |  |
| Down | 37 (47.4) | 28 (47.5) | 1.000 |
| Up | 41 (52.6) | 31 (52.5) |  |
| **NLR change** |  |  |  |
| Down | 58 (63.7) | 41 (63.1) | 1.000 |
| Up | 33 (36.3) | 24 (36.9) |  |
| **PLR change** |  |  |  |
| Down | 55 (59.8) | 40 (61.5) | 0.955 |
| Up | 37 (40.2) | 25 (38.5) |  |
| **LMR change** |  |  |  |
| Down | 56 (60.9) | 34 (52.3) | 0.366 |
| Up | 36 (39.1) | 31 (47.7) |  |
| **SII change** |  |  |  |
| Down | 59 (64.1) | 46 (70.8) | 0.485 |
| Up | 33 (35.9) | 19 (29.2) |  |
| **PNI change** |  |  |  |
| Down | 40 (43.5) | 32 (49.2) | 0.582 |
| Up | 52 (56.5) | 33 (50.8) |  |

ECOG PS, Eastern Cooperative Oncology Group Performance Status. ameans median and interquartile range (IQR), b N (%)

**Supplementary Table 5** Univariable and multivariable Logistic regression model of pPR group patients about systemic inflammation parameters.

| **Variables** | **Univariable** | | **Multivariable** | |
| --- | --- | --- | --- | --- |
|  | OR (95%CI) | P value | OR (95%CI) | P value |
| **Tumor length** | 0.65 (0.47-0.90) | 0.010 | 0.56 (0.32 - 0.97) | **0.037** |
| **Downstaging of N stage** |  |  |  |  |
| No | Ref |  | Ref |  |
| Yes | 8.83 (3.24-24.04) | <.001 | 2.82 (0.75 - 10.59) | 0.126 |
| **Plan** |  |  |  |  |
| ICT | Ref |  | Ref |  |
| CT | 0.47 (0.23-0.95) | 0.036 | 0.93 (0.30 - 2.91) | 0.902 |
| **ECOG** |  |  |  |  |
| 0 | Ref |  |  |  |
| 1 | 1.22 (0.56-2.67) | 0.612 |  |  |
| 2 | 0.88 (0.20-3.86) | 0.866 |  |  |
| **preoperative radiotherapy** |  |  |  |  |
| No | Ref |  | Ref |  |
| Yes | 3.72 (1.49-9.26) | 0.005 | 12.59 (2.76 - 57.48) | **0.001** |
| **Sex** |  |  |  |  |
| Male | Ref |  |  |  |
| Female | 1.10 (0.49-2.46) | 0.811 |  |  |
| **Age** |  |  |  |  |
| <66 | Ref |  |  |  |
| ≥66 | 1.44 (0.76-2.73) | 0.263 |  |  |
| **Tumor location** |  |  |  |  |
| Low | Ref |  |  |  |
| Middle | 0.90 (0.47-1.75) | 0.767 |  |  |
| Upper | 1.13 (0.31-4.08) | 0.851 |  |  |
| **Adverse event** |  |  |  |  |
| No | Ref |  |  |  |
| Yes | 1.37 (0.72-2.62) | 0.335 |  |  |
| **Effect** |  |  |  |  |
| Partial Response | Ref |  | Ref |  |
| Stable Disease | 0.50 (0.26-0.96) | 0.038 | 0.20 (0.07 - 0.61) | 0.005 |
| Progression Disease | 0.00 (0.00-Inf) | 0.988 | 0.00 (0.00 - Inf) | 0.993 |
| **Surgical complications** |  |  |  |  |
| No | Ref |  |  |  |
| Yes | 0.95 (0.46-1.95) | 0.887 |  |  |
| **Differentiation** |  |  |  |  |
| Ⅰ | Ref |  | Ref |  |
| Ⅱ | 0.98 (0.38-2.51) | 0.966 | 0.93 (0.23 - 3.71) | 0.916 |
| Ⅲ | 0.89 (0.31-2.55) | 0.827 | 0.62 (0.13 - 3.07) | 0.557 |
| unknown | 21.33 (2.37-192.03) | 0.006 | 24.92 (1.68 - 369.81) | 0.019 |
| **CEA change** |  |  |  |  |
| Down | Ref |  |  |  |
| Up | 1.00 (0.51-1.97) | 0.998 |  |  |
| **NLR change** |  |  |  |  |
| Down | Ref |  |  |  |
| Up | 1.03 (0.53-1.99) | 0.933 |  |  |
| **PLR change** |  |  |  |  |
| Down | Ref |  |  |  |
| Up | 0.93 (0.48-1.78) | 0.825 |  |  |
| **LMR change** |  |  |  |  |
| Down | Ref |  |  |  |
| Up | 1.42 (0.75-2.70) | 0.286 |  |  |
| **SII change** |  |  |  |  |
| Down | Ref |  |  |  |
| Up | 0.74 (0.37-1.46) | 0.385 |  |  |
| **PNI change** |  |  |  |  |
| Down | Ref |  |  |  |
| Up | 0.79 (0.42-1.50) | 0.476 |  |  |
| **CEA baseline** |  |  |  |  |
| <1.56 | Ref |  | Ref |  |
| ≥1.56 | 0.37 (0.17-0.81) | 0.013 | 0.70 (0.15 - 3.32) | 0.658 |
| **NLR baseline** |  |  |  |  |
| <2.27 | Ref |  |  |  |
| ≥2.27 | 1.64 (0.79-3.38) | 0.181 |  |  |
| **PLR baseline** |  |  |  |  |
| <126.32 | Ref |  |  |  |
| ≥126.32 | 0.70 (0.36-1.37) | 0.296 |  |  |
| **LMR baseline** |  |  |  |  |
| <3.31 | Ref |  |  |  |
| ≥3.31 | 1.64 (0.86-3.11) | 0.133 |  |  |
| **SII baseline** |  |  |  |  |
| <590.55 | Ref |  |  |  |
| ≥590.55 | 0.61 (0.32-1.16) | 0.133 |  |  |
| **PNI baseline** |  |  |  |  |
| <53.23 | Ref |  |  |  |
| ≥53.23 | 0.61 (0.32-1.18) | 0.139 |  |  |
| **CEA 6w** |  |  |  |  |
| <2.16 | Ref |  | Ref |  |
| ≥2.16 | 0.51 (0.25-1.02) | 0.058 | 0.98 (0.25 - 3.86) | 0.979 |
| **NLR 6w** |  |  |  |  |
| <3.36 | Ref |  |  |  |
| ≥3.36 | 1.19 (0.53-2.68) | 0.678 |  |  |
| **PLR 6w** |  |  |  |  |
| <147.45 | Ref |  |  |  |
| ≥147.45 | 0.58 (0.30-1.13) | 0.108 |  |  |
| **LMR 6w** |  |  |  |  |
| <2.14 | Ref |  | Ref |  |
| ≥2.14 | 2.24 (0.93-5.41) | 0.073 | 2.87 (0.68 - 12.17) | 0.153 |
| **SII 6w** |  |  |  |  |
| <265.68 | Ref |  |  |  |
| ≥265.68 | 0.62 (0.30-1.29) | 0.203 |  |  |
| **PNI 6w** |  |  |  |  |
| <51.48 | Ref |  |  |  |
| ≥51.48 | 0.60 (0.32-1.15) | 0.126 |  |  |

**Supplementary Table 6** Univariable and Multivariable Cox regression analysis of Disease-Free Survival about periphery blood systemic inflammation parameters.

| **variable** | **Univariable** | | | **Multivariable** | | |
| --- | --- | --- | --- | --- | --- | --- |
|  | **HR** | **95%CI** | **P value** | **HR** | **95%CI** | **P value** |
| Age | 0.4 | 0.2 - 0.79 | 0.008 | 0.4 | 0.19 - 0.81 | **0.012** |
| Downstaging of N stage | 0.27 | 0.15 - 0.49 | <0.001 | 0.33 | 0.18 - 0.62 | **<0.001** |
| Tumor length | 1.24 | 0.99 - 1.55 | 0.059 | 1.16 | 0.92 - 1.48 | 0.217 |
| PNI change | 0.4 | 0.22 - 0.75 | 0.004 | 0.5 | 0.26 - 0.95 | **0.035** |

**Supplementary Table 7** Univariable and Multivariable Cox regression analysis of Overall survival about periphery blood systemic inflammation parameters.

| **variable** | **Univariable** | | | **Multivariable** | | |
| --- | --- | --- | --- | --- | --- | --- |
|  | **HR** | **95%CI** | **P value** | **HR** | **95%CI** | **P value** |
| Downstaging of N stage | 0.44 | 0.2 - 0.94 | 0.034 | 0.42 | 0.19 - 0.95 | **0.037** |
| Surgical Complications | 2.03 | 0.88 - 4.71 | 0.099 | 1.92 | 0.8 - 4.61 | 0.144 |
| Differentiation | 0.54 | 0.33 - 0.89 | 0.016 | 0.53 | 0.29 - 0.95 | **0.032** |
| Baseline CEA | 7.21 | 0.97 - 53.71 | 0.054 | 4.73 | 0.62 - 36.04 | 0.134 |
| 6W PNI | 2.08 | 0.88 - 4.93 | 0.095 | 2.48 | 1.03 - 5.97 | **0.043** |

**
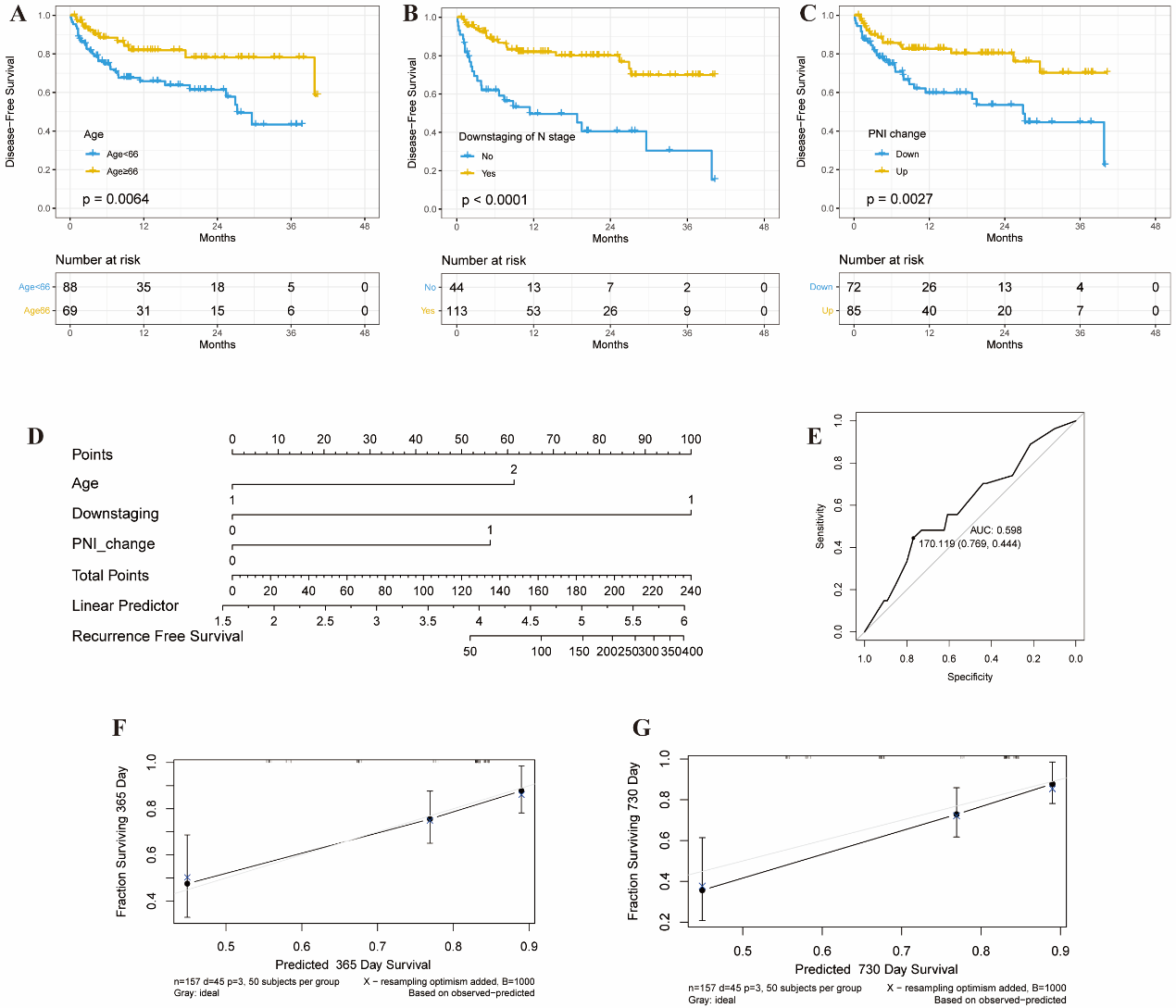
**

**Supplementary Figure 3** The nomogram of DFS in based on peripheral blood inflammatory markers. **A-C** Kaplan–Meier curves related to DFS based on peripheral blood inflammatory markers. **D** Nomogram based on the multivariate model of DFS. **E-G** ROC curves and 365- and 730- days calibration curves.


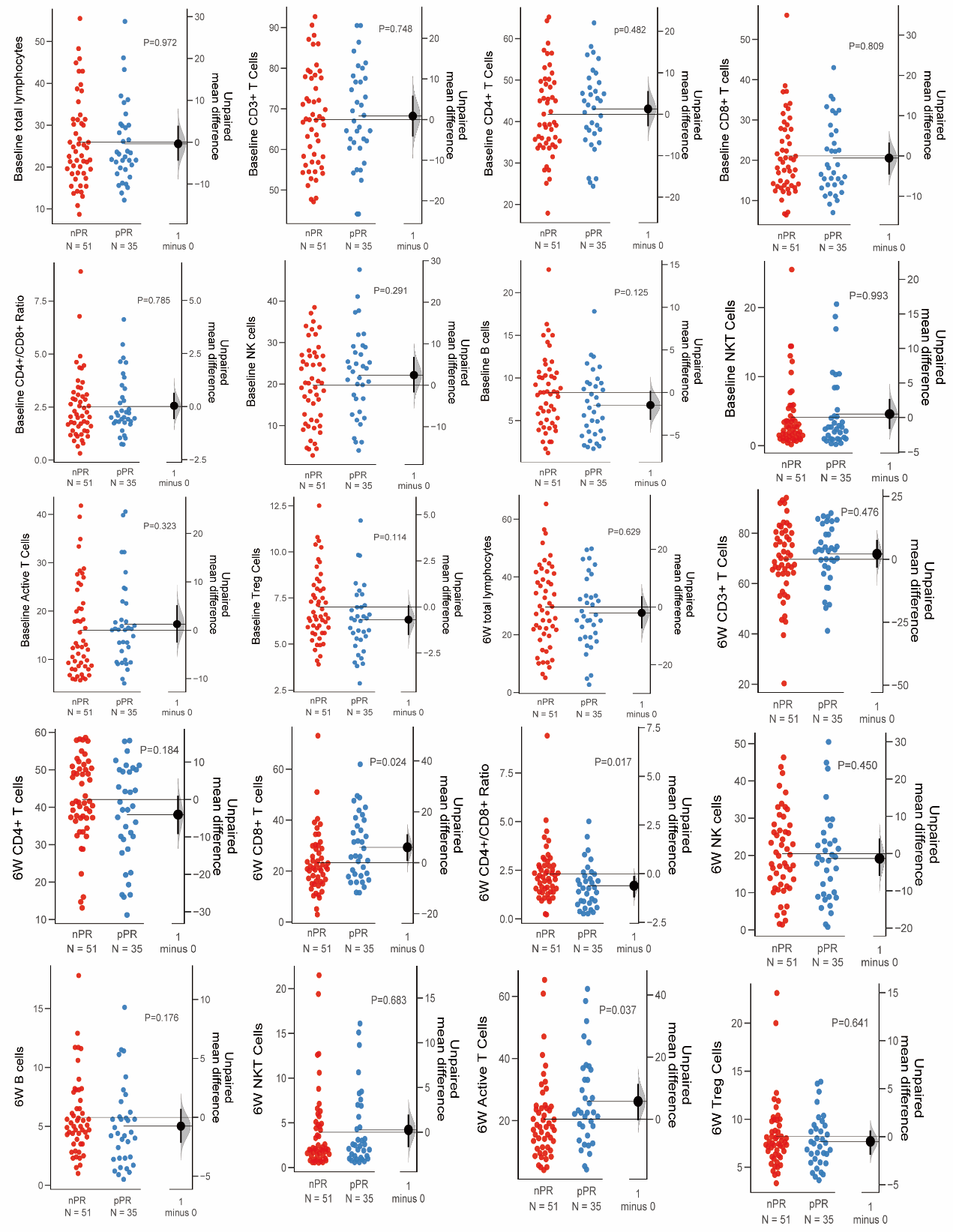


**Supplementary Figure 4** Dynamic changes about peripheral blood lymphocyte subsets between pPR group and nPR group.

**
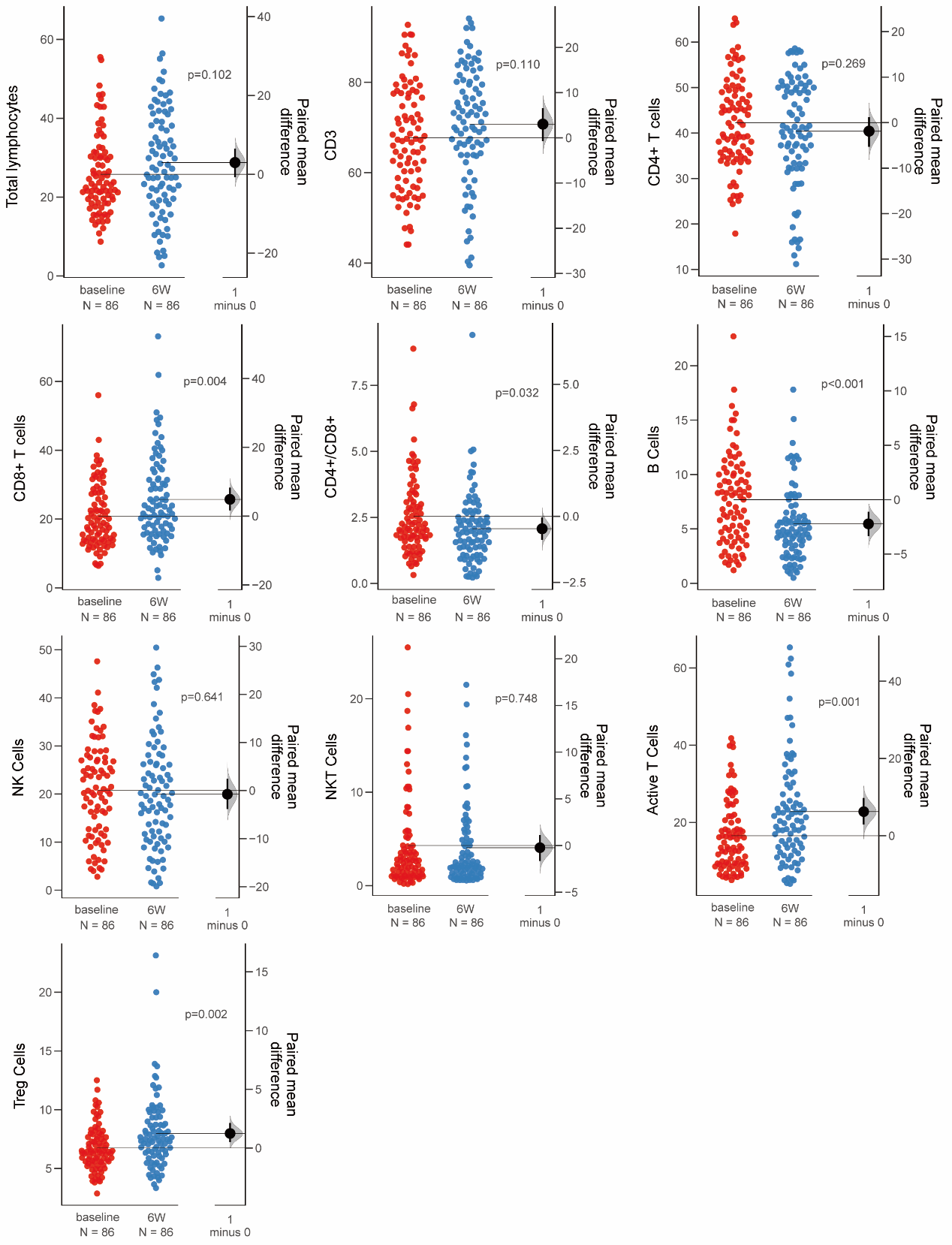
**

**Supplementary Figure 5** Dynamic changes about peripheral blood lymphocyte subsets before and after therapy.

**Supplementary Table 8** The association between periphery lymphocyte subsets and treatment response.

| **Variable** | **nPR (%)** | **pPR (%)** | **P** |
| --- | --- | --- | --- |
| **TLS** |  |  | 0.348 |
| <2.5 | 21 (41.18) | 18 (51.43) |  |
| ≥2.5 | 30 (58.82) | 17 (48.57) |  |
| **Total lymphocytes baseline** |  |  | 0.326 |
| <30.35 | 36 (70.59) | 28 (80.00) |  |
| ≥30.35 | 15 (29.41) | 7 (20.00) |  |
| **CD3+T cells baseline** |  |  | 0.163 |
| <45.6 | 0 (0.00) | 2 (5.71) |  |
| ≥45.6 | 51 (100.00) | 33 (94.29) |  |
| **CD4+T cells baseline** |  |  | 0.112 |
| <36.75 | 20 (39.22) | 8 (22.86) |  |
| ≥36.75 | 31 (60.78) | 27 (77.14) |  |
| **CD8+T cells baseline** |  |  | 0.306 |
| <12.1 | 5 (9.80) | 7 (20.00) |  |
| ≥12.1 | 46 (90.20) | 28 (80.00) |  |
| **CD4+/CD8+ T cells baseline** |  |  | 0.316 |
| <2.43 | 28 (54.90) | 23 (65.71) |  |
| ≥2.43 | 23 (45.10) | 12 (34.29) |  |
| **NK cells baseline** |  |  | 0.174 |
| <20.45 | 28 (54.90) | 14 (40.00) |  |
| ≥20.45 | 23 (45.10) | 21 (60.00) |  |
| **B cells baseline** |  |  | 0.065 |
| <9.95 | 33 (64.71) | 29 (82.86) |  |
| ≥9.95 | 18 (35.29) | 6 (17.14) |  |
| **NKT-like cells baseline** |  |  | 0.171 |
| <8.15 | 45 (88.24) | 27 (77.14) |  |
| ≥8.15 | 6 (11.76) | 8 (22.86) |  |
| **Activated T cells baseline** |  |  | 0.032 |
| <13.42 | 28 (54.90) | 11 (31.43) |  |
| ≥13.42 | 23 (45.10) | 24 (68.57) |  |
| **Treg T cells baseline** |  |  | 0.053 |
| <7.12 | 29 (56.86) | 27 (77.14) |  |
| ≥7.12 | 22 (43.14) | 8 (22.86) |  |
| **Total lymphocyte 6w** |  |  | 0.077 |
| <33.75 | 30 (58.82) | 27 (77.14) |  |
| ≥33.75 | 21 (41.18) | 8 (22.86) |  |
| **CD3^+^T cells 6w** |  |  | 0.085 |
| <68.80 | 24 (47.06) | 10 (28.57) |  |
| ≥68.80 | 27 (52.94) | 25 (71.43) |  |
| **CD4^+^T cells 6w** |  |  | 0.023 |
| <33.3 | 8 (15.69) | 13 (37.14) |  |
| ≥33.3 | 43 (84.31) | 22 (62.86) |  |
| **CD8^+^T cells 6w** |  |  | 0.005 |
| <30.85 | 42 (82.35) | 19 (54.29) |  |
| ≥30.85 | 9 (17.65) | 16 (45.71) |  |
| **CD4^+^/CD8^+^ ratio 6w** |  |  | <0.001 |
| <1.04 | 4 (7.84) | 13 (37.14) |  |
| ≥1.04 | 47 (92.16) | 22 (62.86) |  |
| **NK cells 6w** |  |  | 0.084 |
| <10.3 | 8 (15.69) | 11 (31.43) |  |
| ≥10.3 | 43 (84.31) | 24 (68.57) |  |
| **B cells 6w** |  |  | 0.024 |
| <2.45 | 6 (11.76) | 11 (31.43) |  |
| ≥2.45 | 45 (88.24) | 24 (68.57) |  |
| **NKT-like cells 6w** |  |  | 0.160 |
| <6.75 | 44 (86.27) | 26 (74.29) |  |
| ≥6.75 | 7 (13.73) | 9 (25.71) |  |
| **Activated T cells 6w** |  |  | 0.012 |
| <18.07 | 27 (52.94) | 9 (25.71) |  |
| ≥18.07 | 24 (47.06) | 26 (74.29) |  |
| **Treg T cells 6w** |  |  | 0.222 |
| <6.58 | 14 (27.45) | 14 (40.00) |  |
| ≥6.58 | 37 (72.55) | 21 (60.00) |  |
| **Total lymphocytes change** |  |  | 0.913 |
| Down | 21 (41.18) | 14 (40.00) |  |
| Up | 30 (58.82) | 21 (60.00) |  |
| **CD3^+^T cells change** |  |  | 0.927 |
| Down | 17 (33.33) | 12 (34.29) |  |
| Up | 34 (66.67) | 23 (65.71) |  |
| **CD4^+^T cells change** |  |  | 0.006 |
| Down | 18 (35.29) | 23 (65.71) |  |
| Up | 33 (64.71) | 12 (34.29) |  |
| **CD8^+^T cells change** |  |  | 0.358 |
| Down | 21 (41.18) | 11 (31.43) |  |
| Up | 30 (58.82) | 24 (68.57) |  |
| **CD4^+^/CD8^+^ ratio change** |  |  | 0.403 |
| Down | 32 (62.75) | 25 (71.43) |  |
| Up | 19 (37.25) | 10 (28.57) |  |
| **NK cells change** |  |  | 0.238 |
| Down | 27 (52.94) | 23 (65.71) |  |
| Up | 24 (47.06) | 12 (34.29) |  |
| **B cells change** |  |  | 0.981 |
| Down | 38 (74.51) | 26 (74.29) |  |
| Up | 13 (25.49) | 9 (25.71) |  |
| **NKT-like change** |  |  | 0.877 |
| Down | 30 (58.82) | 20 (57.14) |  |
| Up | 21 (41.18) | 15 (42.86) |  |
| **Activated T cells change** |  |  | 0.026 |
| Down | 22 (43.14) | 7 (20.00) |  |
| Up | 29 (56.86) | 28 (80.00) |  |
| **Treg T cells change** |  |  | 0.578 |
| Down | 19 (37.25) | 11 (31.43) |  |
| Up | 32 (62.75) | 24 (68.57) |  |

**Supplementary Table 9** Univariable and multivariable Logistic regression of pPR about periphery lymphocytes subsets and TLS density.

| **Variables** | **Univariable** | | **Multivariable** | |
| --- | --- | --- | --- | --- |
|  | OR (95%CI) | P value | OR (95%CI) | P value |
| **TLS** | 0.99 (0.84-1.16) | 0.877 |  |  |
| **Total lymphocytes baseline** |  |  |  |  |
| <30.35 | Ref |  |  |  |
| ≥30.35 | 0.60 (0.22-1.67) | 0.328 |  |  |
| **CD3^+^T cells baseline** |  |  |  |  |
| <45.6 | Ref |  |  |  |
| ≥45.6 | 0.00 (0.00-Inf) | 0.992 |  |  |
| **CD4^+^T cells baseline** |  |  |  |  |
| <36.75 | Ref |  |  |  |
| ≥36.75 | 2.18 (0.83-5.74) | 0.115 |  |  |
| **CD8^+^T cells baseline** |  |  |  |  |
| <12.1 | Ref |  |  |  |
| ≥12.1 | 0.43 (0.13-1.50) | 0.188 |  |  |
| **CD4^+^/CD8^+^ ratio baseline** |  |  |  |  |
| <2.43 | Ref |  |  |  |
| ≥2.43 | 0.64 (0.26-1.55) | 0.317 |  |  |
| **NK cells baseline** |  |  |  |  |
| <20.45 | Ref |  |  |  |
| ≥20.45 | 1.83 (0.76-4.37) | 0.176 |  |  |
| **B cells baseline** |  |  |  |  |
| <9.95 | Ref |  | Ref |  |
| ≥9.95 | 0.38 (0.13-1.08) | 0.070 | 0.56 (0.11 - 2.74) | 0.473 |
| **NKT-like cells baseline** |  |  |  |  |
| <8.15 | Ref |  |  |  |
| ≥8.15 | 2.22 (0.70-7.10) | 0.178 |  |  |
| **Activated T cells baseline** |  |  |  |  |
| <13.42 | Ref |  | Ref |  |
| ≥13.42 | 2.66 (1.08-6.55) | 0.034 | 6.99 (1.40 - 34.84) | **0.018** |
| **Treg T cells baseline** |  |  |  |  |
| <7.12 | Ref |  | Ref |  |
| ≥7.12 | 0.39 (0.15-1.02) | 0.056 | 0.22 (0.06 - 0.89) | **0.033** |
| **Total lymphocyte 6w** |  |  |  |  |
| <33.75 | Ref |  | Ref |  |
| ≥33.75 | 0.42 (0.16-1.11) | 0.081 | 0.26 (0.07 - 0.99) | **0.048** |
| **CD3^+^T cells 6w** |  |  |  |  |
| <68.80 | Ref |  | Ref |  |
| ≥68.80 | 2.22 (0.89-5.56) | 0.088 | 4.01 (0.96 - 16.79) | 0.057 |
| **CD4^+^T cells 6w** |  |  |  |  |
| <33.3 | Ref |  | Ref |  |
| ≥33.3 | 0.31 (0.11-0.87) | 0.026 | 0.63 (0.10 - 3.78) | 0.613 |
| **CD8^+^T cells 6w** |  |  |  |  |
| <30.85 | Ref |  | Ref |  |
| ≥30.85 | 3.93 (1.47-10.47) | 0.006 | 0.09 (0.01 - 1.01) | 0.051 |
| **CD4^+^/CD8^+^ ratio 6w** |  |  |  |  |
| <1.04 | Ref |  | Ref |  |
| ≥1.04 | 0.14 (0.04-0.49) | 0.002 | 0.07 (0.00 - 0.93) | **0.044** |
| **NK cells 6w** |  |  |  |  |
| <10.3 | Ref |  | Ref |  |
| ≥10.3 | 0.41 (0.14-1.15) | 0.089 | 0.31 (0.06 - 1.61) | 0.164 |
| **B cells 6w** |  |  |  |  |
| <2.45 | Ref |  | Ref |  |
| ≥2.45 | 0.29 (0.10-0.88) | 0.029 | 1.55 (0.26 - 9.13) | 0.631 |
| **NKT-like cells 6w** |  |  |  |  |
| <6.75 | Ref |  |  |  |
| ≥6.75 | 2.18 (0.72-6.54) | 0.166 |  |  |
| **Activated T cells 6w** |  |  |  |  |
| <18.07 | Ref |  | Ref |  |
| ≥18.07 | 3.25 (1.27-8.29) | 0.014 | 0.88 (0.19 - 4.16) | 0.876 |
| **Treg cells 6w** |  |  |  |  |
| <6.58 | Ref |  |  |  |
| ≥6.58 | 0.57 (0.23-1.42) | 0.225 |  |  |
| **Total lymphocytes change** |  |  |  |  |
| Down | Ref |  |  |  |
| Up | 1.05 (0.44-2.52) | 0.913 |  |  |
| **CD3^+^T cells change** |  |  |  |  |
| Down | Ref |  |  |  |
| Up | 0.96 (0.39-2.38) | 0.927 |  |  |
| **CD4^+^T cells change** |  |  |  |  |
| Down | Ref |  | Ref |  |
| Up | 0.28 (0.12-0.70) | 0.006 | 0.28 (0.07 - 1.14) | 0.075 |
| **CD8^+^T cells change** |  |  |  |  |
| Down | Ref |  |  |  |
| Up | 1.53 (0.62-3.78) | 0.359 |  |  |
| **CD4^+^/CD8^+^ ratio change** |  |  |  |  |
| Down | Ref |  |  |  |
| Up | 0.67 (0.27-1.70) | 0.404 |  |  |
| **NK cells change** |  |  |  |  |
| Down | Ref |  |  |  |
| Up | 0.59 (0.24-1.43) | 0.240 |  |  |
| **B cells change** |  |  |  |  |
| Down | Ref |  |  |  |
| Up | 1.01 (0.38-2.71) | 0.981 |  |  |
| **NKT-like change** |  |  |  |  |
| Down | Ref |  |  |  |
| Up | 1.07 (0.45-2.56) | 0.877 |  |  |
| **Activated T cells change** |  |  |  |  |
| Down | Ref |  | Ref |  |
| Up | 3.03 (1.12-8.22) | 0.029 | 4.40 (0.93 - 20.83) | 0.062 |
| **Treg cells change** |  |  |  |  |
| Down | Ref |  |  |  |
| Up | 1.30 (0.52-3.22) | 0.578 |  |  |

**Supplementary Table 10** Univariable and Multivariable Cox regression analysis of Disease-Free Survival about periphery lymphocytes subsets and TLS density.

| **variable** | **Univariable** | | | **Multivariable** | | |
| --- | --- | --- | --- | --- | --- | --- |
|  | **HR** | **95%CI** | **P value** | **HR** | **95%CI** | **P value** |
| Age | 0.42 | 0.18 - 0.98 | 0.046 | 0.4 | 0.16 - 0.96 | **0.040** |
| Downstaging of N stage | 0.21 | 0.09 - 0.48 | <0.001 | 0.23 | 0.09 - 0.54 | **0.001** |
| CD4^+^/CD8^+^ ratio change | 2.37 | 1.13 - 4.98 | 0.023 | 2.4 | 1.11 - 5.17 | **0.026** |
| NK change | 0.46 | 0.2 - 1.06 | 0.069 | 0.36 | 0.14 - 0.90 | **0.028** |
| CD4^+^ T cells baseline | 0.37 | 0.18 - 0.8 | 0.011 | 0.63 | 0.28 - 1.41 | 0.265 |

**Supplementary Table 11** Univariable and Multivariable Cox regression analysis of Overall survival about periphery lymphocytes subsets and TLS density.

| **variable** | **Univariable** | | | **Multivariable** | | |
| --- | --- | --- | --- | --- | --- | --- |
|  | **HR** | **95%CI** | **P value** | **HR** | **95%CI** | **P value** |
| Downstaging of N stage | 0.26 | 0.08 - 0.83 | 0.023 | 0.13 | 0.03 - 0.5 | **0.003** |
| Surgical Complications | 3.26 | 0.97 - 10.96 | 0.056 | 8.02 | 1.77 - 36.29 | **0.007** |
| CD4^+^ T cells change | 2.92 | 0.91 - 9.35 | 0.072 | 2.27 | 0.65 - 7.91 | 0.197 |
| NK cells change | 0.3 | 0.08 - 1.07 | 0.064 | 0.21 | 0.05 - 0.94 | **0.041** |
| TLS | 1.15 | 0.98 - 1.36 | 0.095 | 1.25 | 1.02 - 1.53 | **0.033** |

**
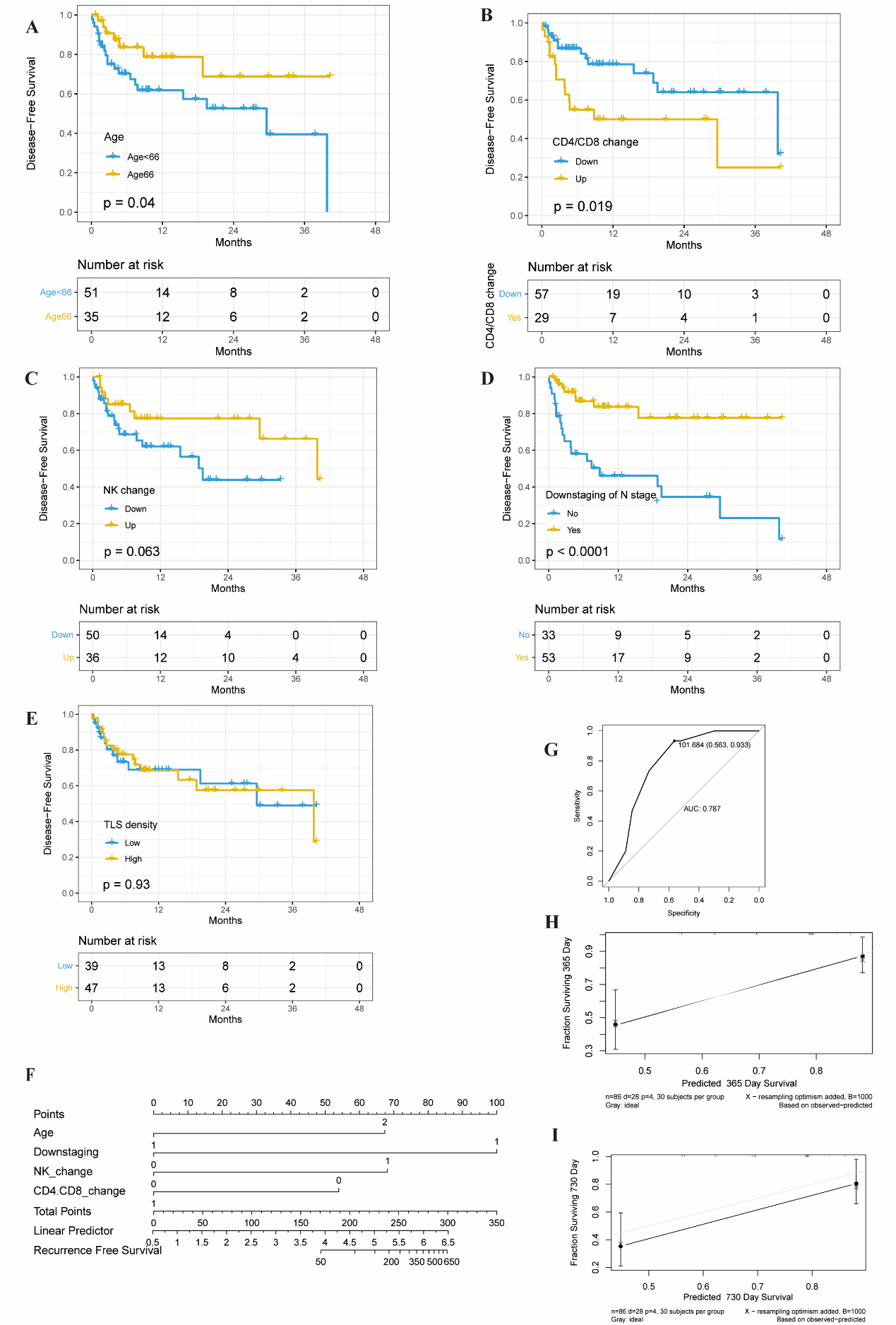
**

**Supplementary Figure 6** The nomogram of DFS in based on peripheral blood lymphocyte subsets. **A-E** Kaplan–Meier curves related to DFS based on lymphocyte subsets. **F** Nomogram based on the multivariate model of DFS. **G-I** ROC curves and 365- and 730- days calibration curves.

**
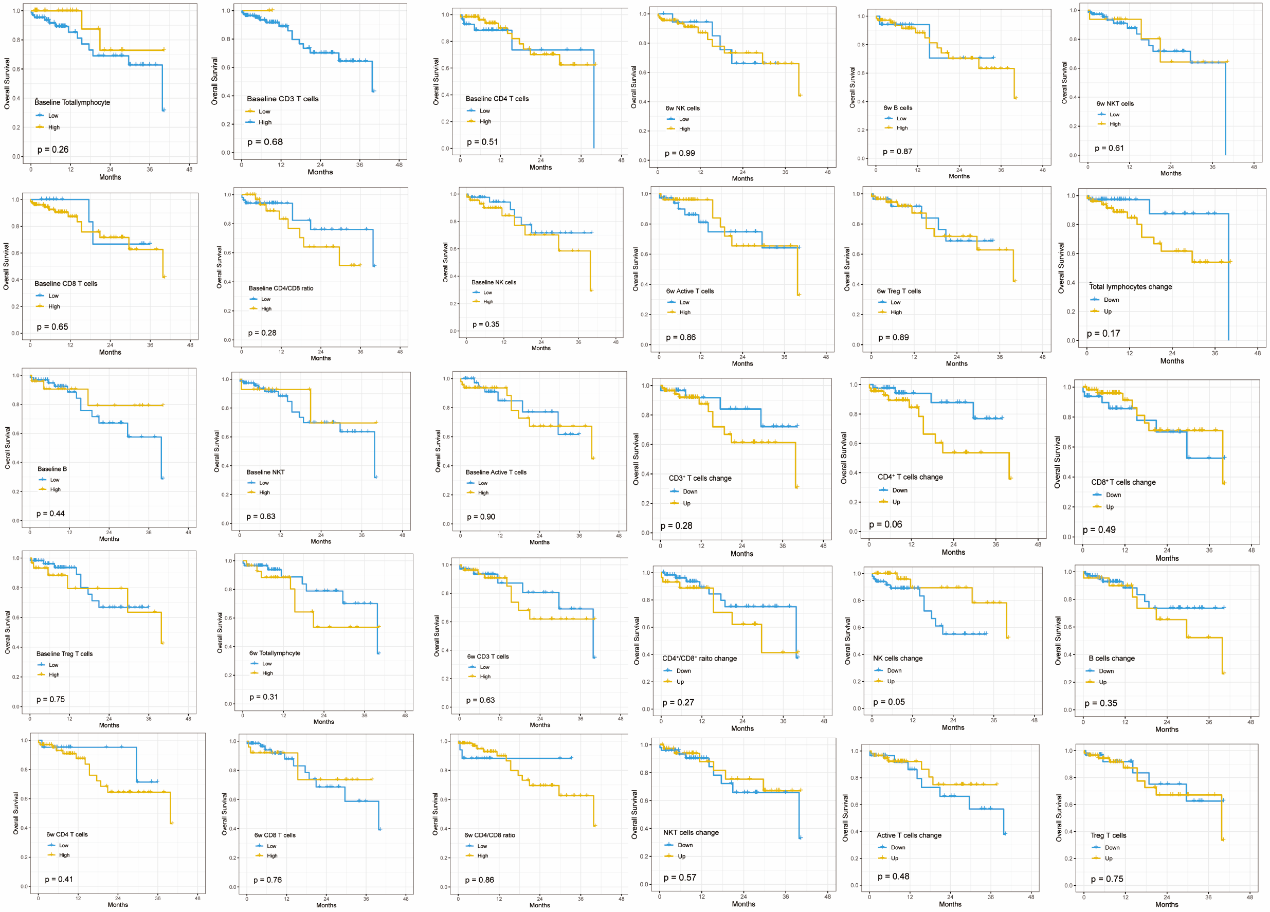
**

**Supplementary Figure 7** Relationship between lymphocytes subsets and OS. The survival curve was plotted using the Kaplan–Meier method and the log rank test was used to determine statistical significance; p<0.05 was considered statistically significant.

**
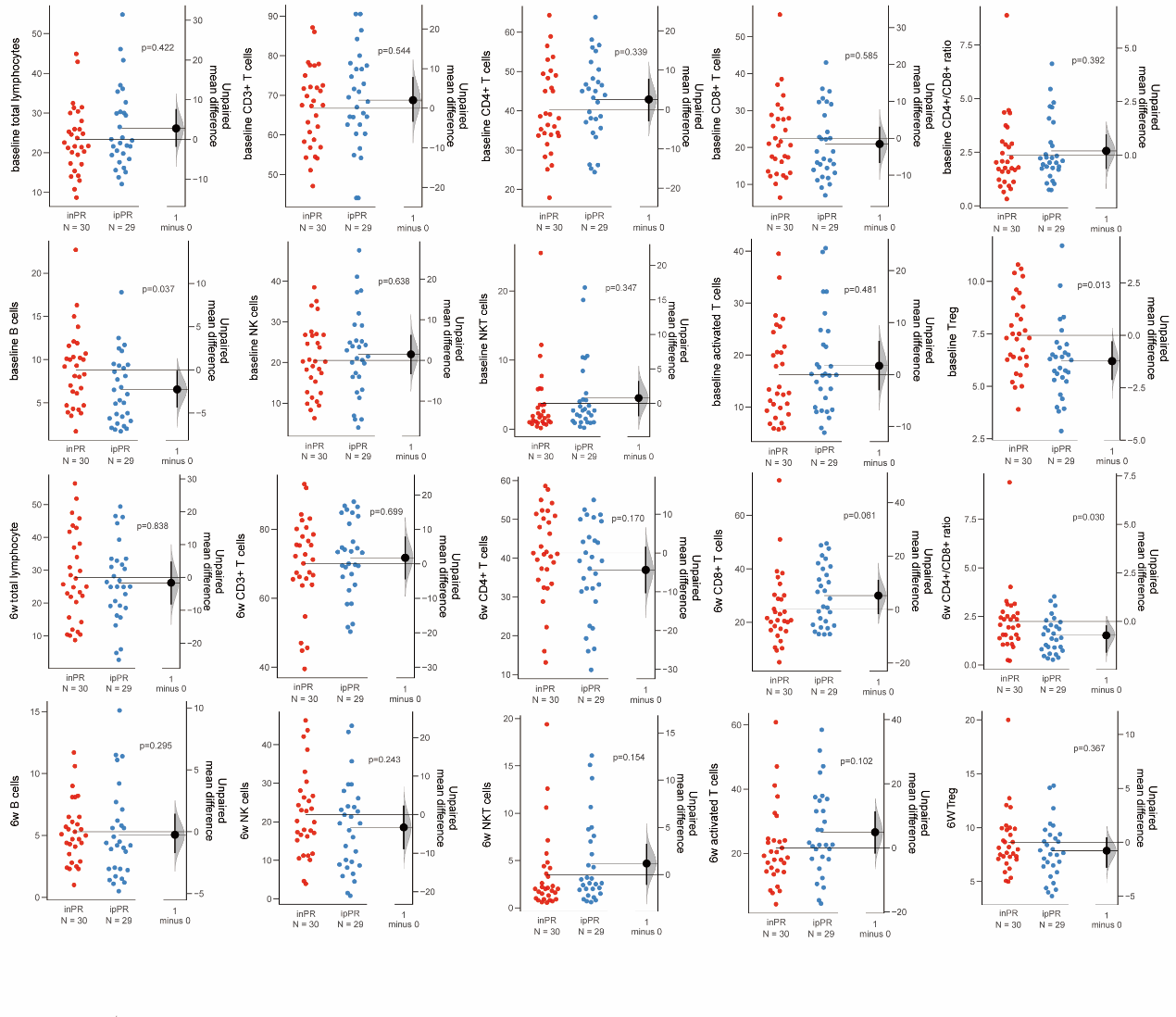
**

**Supplementary Figure 8** Dynamic changes in peripheral lymphocyte subsets were observed in patients receiving immunotherapy. The figure shows the differences between ipPR and inPR groups.

**
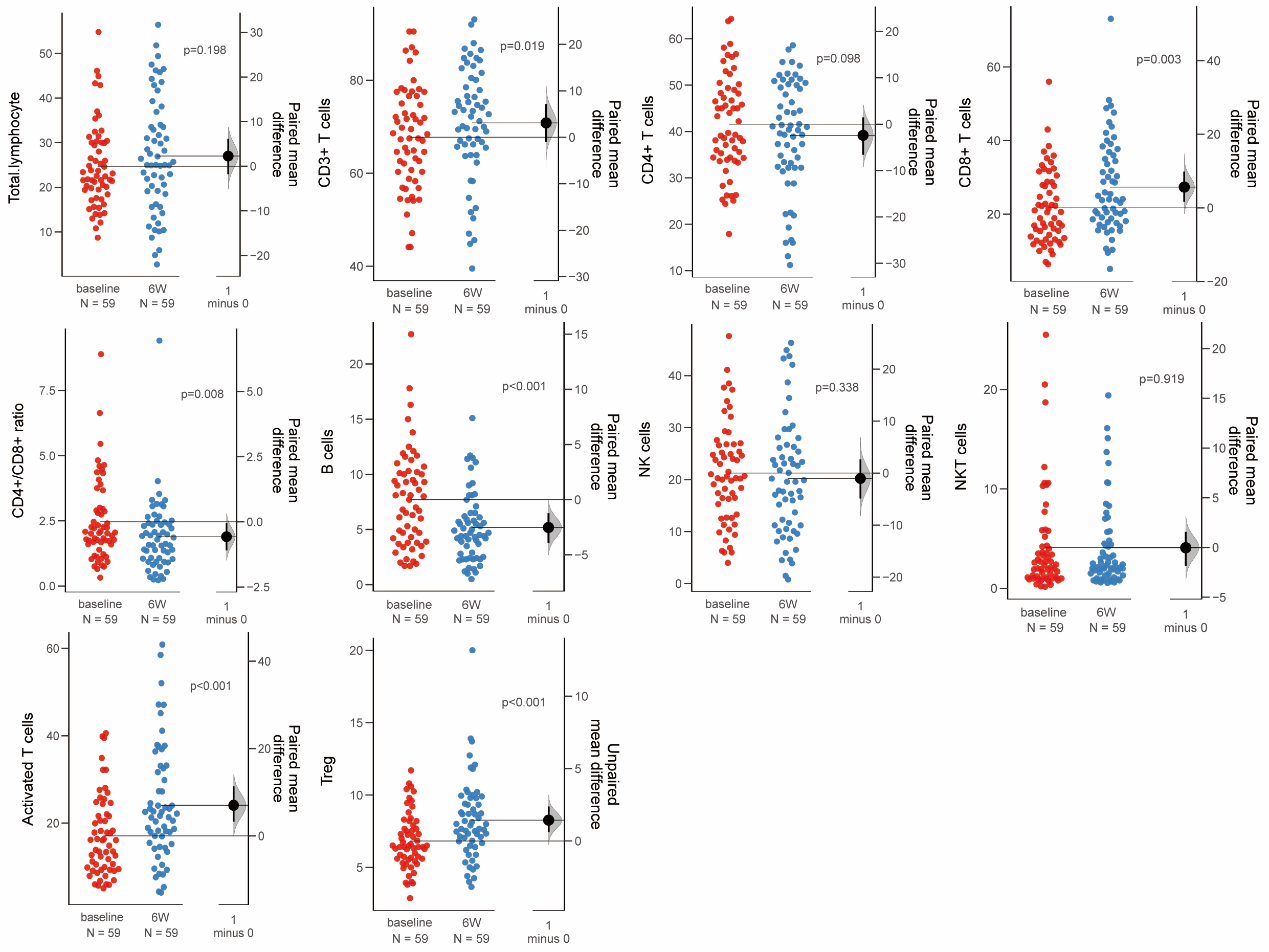
**

**Supplementary Figure 9** Dynamic changes in peripheral lymphocyte subsets were observed in patients receiving immunotherapy before and after therapy.

**Supplementary Table 12** Multivariable Logistic regression model of pPR group in immunotherapy patients (ipPR) about periphery blood systemic inflammation parameters.

| **Variables** | **β** | **S.E** | **Z** | **OR (95%CI)** | **P value** |
| --- | --- | --- | --- | --- | --- |
| **Tumor length** | -0.80 | 0.35 | -2.25 | 0.45 (0.23 - 0.90) | **0.024** |
| **Differentiation** |  |  |  |  |  |
| Ⅰ |  |  |  | Ref |  |
| Ⅱ | 1.32 | 0.90 | 1.47 | 3.73 (0.64 - 21.68) | 0.143 |
| Ⅲ | 1.29 | 1.06 | 1.21 | 3.63 (0.45 - 29.25) | 0.225 |
| unknown | 3.89 | 1.65 | 2.35 | 48.71 (1.92 - 1236.64) | 0.019 |
| **Effect** |  |  |  |  |  |
| Partial Response |  |  |  | Ref |  |
| Stable Disease | -1.00 | 0.62 | -1.61 | 0.37 (0.11 - 1.24) | 0.107 |
| Progression Disease | -9.01 | 1455.40 | -0.01 | 0.00 (0.00 - Inf) | 0.995 |
| **Downstaging of N stage** |  |  |  |  |  |
| No |  |  |  | Ref |  |
| Yes | 3.15 | 1.21 | 2.60 | 23.26 (2.17 - 249.72) | **0.009** |
| **CEA Baseline** |  |  |  |  |  |
| <2.12 |  |  |  | Ref |  |
| ≥2.12 | -0.79 | 1.00 | -0.79 | 0.46 (0.06 - 3.20) | 0.429 |
| **CEA 6W** |  |  |  |  |  |
| <2.18 |  |  |  | Ref |  |
| ≥2.18 | -0.62 | 0.92 | -0.67 | 0.54 (0.09 - 3.24) | 0.500 |
| **PNI 6W** |  |  |  |  |  |
| <51.50 |  |  |  | Ref |  |
| ≥51.50 | -1.61 | 0.74 | -2.16 | 0.20 (0.05 - 0.86) | **0.031** |

**Supplementary Table 13** Multivariable Logistic regression of ipPR about periphery lymphocytes subsets and TLS density.

| **Variables** | **β** | **S.E** | **Z** | **OR (95%CI)** | **P value** |
| --- | --- | --- | --- | --- | --- |
| **CD4^+^T cells change** |  |  |  |  |  |
| Down |  |  |  | Ref |  |
| Up | -0.69 | 1.21 | -0.57 | 0.50 (0.05 - 5.40) | 0.571 |
| **CD8^+^T cells change** |  |  |  |  |  |
| Down |  |  |  | Ref |  |
| Up | 2.41 | 1.23 | 1.97 | 11.14 (1.01 - 123.02) | **0.049** |
| **Activated T cells change** |  |  |  |  |  |
| Down |  |  |  | Ref |  |
| Up | 1.14 | 1.35 | 0.84 | 3.12 (0.22 - 44.39) | 0.400 |
| **CD4^+^T cells baseline** |  |  |  |  |  |
| <39.50 |  |  |  | Ref |  |
| ≥39.50 | 4.15 | 1.89 | 2.19 | 63.27 (1.55 - 2578.44) | **0.028** |
| **B cells baseline** |  |  |  |  |  |
| <3.85 |  |  |  | Ref |  |
| ≥3.85 | -0.85 | 1.45 | -0.59 | 0.43 (0.02 - 7.30) | 0.556 |
| **Activated T cells baseline** |  |  |  |  |  |
| <13.42 |  |  |  | Ref |  |
| ≥13.42 | 3.64 | 1.67 | 2.17 | 37.98 (1.43 - 1006.78) | **0.030** |
| **Treg T cells baseline** |  |  |  |  |  |
| <5.97 |  |  |  | Ref |  |
| ≥5.97 | -3.39 | 1.58 | -2.14 | 0.03 (0.00 - 0.75) | **0.032** |
| **CD8^+^T cells 6w** |  |  |  |  |  |
| <30.35 |  |  |  | Ref |  |
| ≥30.35 | -2.03 | 1.41 | -2.06 | 0.13 (0.00 – 2.10) | 0.151 |
| **CD4^+^/CD8^+^ ratio 6w** |  |  |  |  |  |
| <2.31 |  |  |  | Ref |  |
| ≥2.31 | -4.87 | 2.44 | -2.00 | 0.01 (0.00 - 0.91) | **0.046** |
| **NK cells 6w** |  |  |  |  |  |
| <18.65 |  |  |  | Ref |  |
| ≥18.65 | -0.73 | 1.23 | -0.59 | 0.48 (0.04 - 5.40) | 0.553 |
| **B cells 6w** |  |  |  |  |  |
| <4.25 |  |  |  | Ref |  |
| ≥4.25 | 1.92 | 1.51 | 1.27 | 6.83 (0.35 - 132.80) | 0.205 |
| **Activated T cells 6w** |  |  |  |  |  |
| <21.17 |  |  |  | Ref |  |
| ≥21.17 | 1.15 | 1.43 | 0.80 | 3.15 (0.19 - 52.38) | 0.424 |

**Supplementary Table 14** Univariable and Multivariable Cox regression analysis of Disease-Free Survival about periphery blood systemic inflammation parameters in ipPR group.

| **variable** | **Univariable** | | | **Multivariable** | | |
| --- | --- | --- | --- | --- | --- | --- |
|  | **HR** | **95%CI** | **P value** | **HR** | **95%CI** | **P value** |
| Age | 0.26 | 0.1 - 0.7 | 0.007 | 0.22 | 0.08 - 0.6 | **0.003** |
| Downstaging of N stage | 0.27 | 0.13 - 0.57 | 0.001 | 0.35 | 0.16 - 0.74 | **0.006** |
| PNI change | 0.28 | 0.13 - 0.62 | 0.002 | 0.3 | 0.13 - 0.69 | **0.004** |
| PLR 6W | 1.88 | 0.9 - 3.92 | 0.093 | 1.48 | 0.69 - 3.18 | 0.312 |

**Supplementary Table 15** Univariable and Multivariable Cox regression analysis of Overall survival about periphery blood systemic inflammation parameters in ipPR group.

| **variable** | **Univariable** | | | **Multivariable** | | |
| --- | --- | --- | --- | --- | --- | --- |
|  | **HR** | **95%CI** | **P value** | **HR** | **95%CI** | **P value** |
| Age | 0.24 | 0.05 - 1.07 | 0.061 | 0.28 | 0.06 - 1.25 | 0.094 |
| Downstaging of N stage | 0.31 | 0.11 - 0.89 | 0.030 | 0.29 | 0.1 - 0.89 | **0.030** |
| Differentiation | 0.55 | 0.29 - 1.05 | 0.071 | 0.55 | 0.28 - 1.09 | 0.085 |

**Supplementary Table 16** Univariable and Multivariable Cox regression analysis of Disease-Free Survival about periphery lymphocytes subsets and TLS density in ipPR group

| **variable** | **Univariable** | | | **Multivariable** | | |
| --- | --- | --- | --- | --- | --- | --- |
|  | **HR** | **95%CI** | **P value** | **HR** | **95%CI** | **P value** |
| Age | 0.26 | 0.08 - 0.92 | 0.037 | 0.25 | 0.07 - 0.92 | **0.037** |
| Downstaging of N stage | 0.18 | 0.07 - 0.48 | 0.001 | 0.26 | 0.09 - 0.74 | **0.012** |
| CD4^+^/CD8^+^ ratio change | 3.13 | 1.21 - 8.09 | 0.019 | 3.32 | 1.19 - 9.26 | **0.022** |
| CD4^+^ T cells baseline | 0.29 | 0.11 - 0.76 | 0.012 | 0.39 | 0.14 - 1.05 | 0.063 |

**Supplementary Table 17** Univariable and Multivariable Cox regression analysis of Overall survival about periphery lymphocytes subsets and TLS density in ipPR group.

| **variable** | **Univariable** | | | **Multivariable** | | |
| --- | --- | --- | --- | --- | --- | --- |
|  | **HR** | **95%CI** | **P value** | **HR** | **95%CI** | **P value** |
| Downstaging of N stage | 0.15 | 0.03 - 0.78 | 0.023 | 0.12 | 0.02 – 0.83 | **0.032** |
| Surgical Complications | 4.22 | 0.89 - 20.08 | 0.071 | 91.81 | 2.52 – 3343.98 | **0.014** |
| Tumor length | 1.85 | 1.06 - 3.23 | 0.03 | 2.3 | 0.68 - 7.83 | 0.181 |
| TLS | 1.23 | 0.96 - 1.58 | 0.098 | 1.98 | 0.8 - 4.94 | 0.141 |
| CD4^+^/CD8^+^ ratio change | 3.41 | 0.8 - 14.45 | 0.097 | 2.89 | 0.31 - 27.34 | 0.354 |
| B cells change | 5.2 | 1.02 - 26.6 | 0.048 | 27.77 | 1.47 – 526.21 | **0.027** |


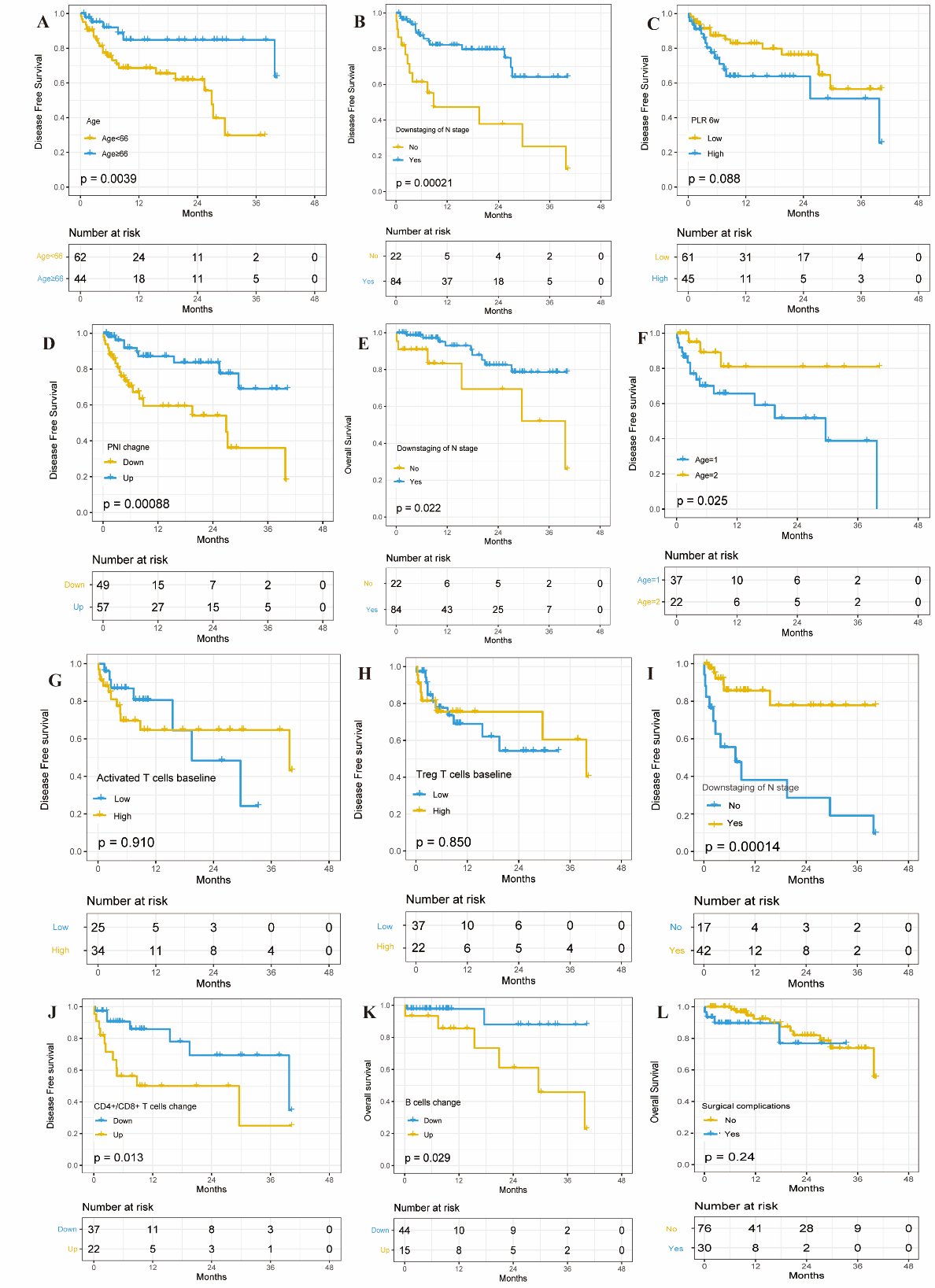


**Supplementary Figure 10** **A-E** KM curves of predictive indicators for DFS and OS about inflammatory markers in patients receiving neo-adjuvant immunotherapy. **F-L** KM curves for DFS or OS about lymphocyte subgroups. The log rank test was used to determine statistical significance; p<0.05 was considered statistically significant.
